# Multidisciplinary monitoring and stakeholder engagement support large carnivore restoration in human‐dominated landscape

**DOI:** 10.1002/eap.70052

**Published:** 2025-05-26

**Authors:** Miha Krofel, Urša Fležar, Rok Černe, Lan Hočevar, Marjeta Konec, Aleksandra Majić Skrbinšek, Tomaž Skrbinšek, Seth Wilson, Bernarda Bele, Jaka Črtalič, Tomislav Gomerčić, Tilen Hvala, Jakub Kubala, Pavel Kvapil, Meta Mavec, Anja Molinari‐Jobin, Paolo Molinari, Elena Pazhenkova, Hubert Potočnik, Teodora Sin, Magda Sindičić, Ira Topličanec, Teresa Oliveira

**Affiliations:** ^1^ Biotechnical Faculty University of Ljubljana Ljubljana Slovenia; ^2^ IUCN SSC Cat Specialist Group Gland Switzerland; ^3^ Slovenia Forest Service Ljubljana Slovenia; ^4^ DivjaLabs Ltd. Ljubljana Slovenia; ^5^ Blackfoot Challenge Ovando Montana USA; ^6^ Faculty of Veterinary Medicine University of Zagreb Zagreb Croatia; ^7^ Hunters Association of Slovenia Ljubljana Slovenia; ^8^ Technical University in Zvolen Zvolen Slovakia; ^9^ Ljubljana ZOO Ljubljana Slovenia; ^10^ Progetto Lince Italia Tarvisio Italy; ^11^ Association for the Conservation of Biological Diversity Focsani Romania

**Keywords:** camera trapping, Eurasian lynx, inbreeding, *Lynx lynx*, public attitudes, reinforcement, reintroduction, survival, translocation

## Abstract

Translocations are central to large carnivore restoration efforts, but inadequate monitoring often inhibits effective conservation decision‐making. Extinctions, reintroductions, illegal killings, and high inbreeding levels of the Central European populations of Eurasian lynx (*Lynx lynx*) typify the carnivore conservation challenges in the Anthropocene. Recently, several conservation efforts were initiated to improve the genetic and demographic status but were met with variable success. Here, we report on successful, stakeholder‐engaged translocation efforts across three countries aimed to: (1) reinforce the Dinaric lynx population that was suffering from high inbreeding levels and (2) create a new stepping‐stone subpopulation in the neighboring Southeastern Alps to help connect the Dinaric and Alpine populations. To evaluate the success of these efforts, we used multidisciplinary and internationally coordinated monitoring using systematic camera trapping, non‐invasive genetic sampling, GPS tracking, recording of reproductive events and interspecific interactions, as well as the simultaneous tracking of the public and stakeholders' support of lynx conservation before, during, and after the translocations. Among the 22 translocated wild‐caught Carpathian lynx, 68% successfully integrated into the population and local ecosystems, and at least 59% reproduced. The probability of dispersing from the release areas was three times lower with the soft‐release method than with hard‐release method. Translocated individuals had substantially lower natural mortality and higher reproductive success, while their ecological impact was similar compared to the lynx from the remnant population. Cooperation with local hunters and protected area managers enabled us to conduct multi‐year camera‐trapping and non‐invasive genetic monitoring across a 12,000‐km^2^ transboundary area. Results indicate a reversal in population decline, as the lynx abundance increased for >40% during the 4‐year translocation period. Effective inbreeding decreased from 0.32 to 0.08–0.19, suggesting a twofold to fourfold increase in fitness. Furthermore, the successful establishment of a new stepping‐stone subpopulation represents an important step toward restoring the Central European lynx metapopulation. Robust partnerships with local communities and hunters, coupled with transparent communication, helped maintain high public and stakeholder support for lynx conservation throughout the translocation efforts. Lessons learned about the importance of stakeholder involvement and multidisciplinary monitoring conducted across several countries provide a successful example for further efforts to restore large carnivores in human‐dominated ecosystems.

## INTRODUCTION

Large carnivores can play a key role in the structure and function of ecosystems (Ripple et al., 2014). However, they are disproportionately affected by humans due to their large habitat needs, apex trophic position, frequent conflicts with people, and high levels of human‐caused mortality (Darimont et al., 2015; Krofel et al., 2015; Ripple et al., 2014). To counteract declines of large carnivore populations and restore functional ecosystems, conservationists and managers increasingly use translocations to reintroduce extirpated species to their historic ranges or to reinforce declining populations by adding individuals from another population or captive breeding programs (Thomas et al., 2023). Although survivorship of translocated large carnivores has improved in the last decades and it is higher than other vertebrates, only 66% of individuals survive the first 6 months and 37% reproduce (Thomas et al., 2023). This necessitates a better and more holistic understanding of the translocation efforts to enable evidence‐based and adaptive decision‐making (Taylor et al., 2017).

Successful large carnivore translocations must consider both biological and social factors that are context specific to the species, ecosystems, cultural context, and landscapes where large carnivore recovery efforts take place. These include choosing optimal individual animals and release sites, deciding whether to keep animals for an acclimation period at the release site (i.e., soft‐release) or release them without an acclimation period (i.e., hard‐release) (Thomas et al., 2023), assessing the likelihood of successful integration into local ecosystems through predation and other interspecific interactions (Hayward & Somers, 2009), and understanding public attitudes and support among key stakeholder groups (Wilson, 2018). Many past monitoring efforts have been limited to tracking the movement of translocated animals and have paid less attention to the broader population and ecological impacts or how the translocation influenced public and stakeholder attitudes (Taylor et al., 2017; Wilson, 2018). Large carnivores are among the most conflict‐prone species, and their translocations often result in controversies and public opposition, making it as much a political as a biological challenge (Treves & Karanth, 2003). Understanding public attitudes, which can influence human behavior (Thorn et al., 2015), is an essential part of any reinforcement or reintroduction effort, and this is especially relevant in human‐dominated landscapes, like Central Europe, where the scale of large carnivore populations transcends national borders, cultures, and management jurisdictions (Penteriani et al., 2018).

The Eurasian lynx (*Lynx lynx*; hereafter lynx) is an apex predator specialized in hunting wild ungulates and is considered a species of conservation concern in Europe (von Arx et al., 2021). While large, autochthonous populations persist in Northern and Eastern Europe, lynx were exterminated from Western and Central Europe by the beginning of the 20th century. Since the 1970s, several reintroduction attempts have been made, some of which restored lynx to portions of their historic ranges (Linnell et al., 2009). However, these reintroduced populations remain isolated and are currently all classified as endangered or critically endangered (von Arx et al., 2021). These populations are primarily threatened by inbreeding depression (Mueller et al., 2022) and illegal killing, resulting from low acceptance by certain members of local hunting communities that perceive lynx as competitors for wild ungulates, as well as due to other, deeper‐rooted traditional motives (Heurich et al., 2018; Premier et al., 2025). Recently, several conservation efforts were initiated to improve the genetic and demographic status of these lynx populations through reinforcement of the existing populations or creating new stepping‐stone subpopulations (i.e., small lynx occurrences that potentially connect otherwise isolated populations) to promote gene flow and resilience (Molinari et al., 2021; Port et al., 2024).

The Dinaric lynx population was considered the most inbred lynx population in Europe (Mueller et al., 2022; Sindičić et al., 2013). This population originated from the 1973 translocation of six individuals (some of which were related to one another) from the Carpathian population to the Dinaric Mountains of Slovenia. Although initially the effort was successful as lynx reproduced and expanded their population to neighboring countries along the Dinaric Mountains (Croatia and Bosnia and Herzegovina) and the Southeastern Alps (Italy and Austria), by the 1990s the population expansion stalled and rapidly declined after the 2000s (Fležar et al., 2021). By 2019, lynx were functionally extinct in the Southeastern Alps, and the remnant population in the Dinaric Mountains was highly inbred (Fe > 0.3), showed signs of inbreeding depression, and faced immediate extinction risk (Fležar et al., 2021; Molinari et al., 2021; Pazhenkova et al., 2025; Sindičić et al., 2013).

Following favorable public attitude surveys supportive of lynx conservation (Majić Skrbinšek, 2008), several international workshops were convened by leading experts to develop a reinforcement in the 2010s (Breitenmoser, 2011; Krofel et al., 2010). Based on an agreement that wild‐caught lynx from the Carpathian population would be the most suitable source of lynx for the reinforcement project, the European Union (EU)‐funded LIFE Lynx project started in 2017. During the first 2 years of the project, LIFE Lynx project team members in Slovenia, Croatia, and Italy engaged in planning, outreach, education, and meaningful engagement with the public, local communities, and hunters to build support for the translocations (for details, see *Maintaining public and hunter support*). We identified hunters as the key stakeholders in the region due to their crucial role in wildlife management, conservation, and monitoring (Fležar et al., 2025). At the same time, hunters also present a potential risk, as some individuals may engage in illegal killing, which presents the main mortality cause for lynx across Europe (Heurich et al., 2018; Premier et al., 2025). This preparatory work was followed by the translocation of 18 lynx from the Carpathian population (Romania and Slovakia) to the Dinaric Mountains (Slovenia and Croatia) and Southeastern Alps (Slovenia) in 2019–2023. An additional four lynx were translocated from the Carpathian (Romania) and Jura populations (Switzerland) to the Southeastern Alps (Italy) in 2023 in the frame of the Urgent Lynx Conservation Action (ULyCA2) project.

These two projects shared three main goals: (1) use population reinforcement to prevent the extinction of the Dinaric population, (2) reintroduce lynx to the Julian Alps to create a new stepping‐stone subpopulation in the Southeastern Alps to facilitate connection between the Dinaric and (Western) Alpine populations, and (3) maintain support for lynx conservation among the public and key stakeholders (hunters) through strategic communication and strong partnerships.

We report on the results of these conservation efforts in the Dinaric Mountains and the Southeastern Alps, including the integration of the translocated lynx into the population and local ecosystems, as well as the efficacy of different release methods (soft vs. hard releases). We also compared survival, mortality causes, reproduction, and foraging among translocated lynx and lynx from the remnant population. In parallel, we conducted systematic camera‐trapping and non‐invasive genetic monitoring of the population at a transboundary level to continuously measure the population‐level impact of translocations on demography and genetic diversity. Results of this monitoring guided adaptive decision‐making on the annual basis throughout the translocation efforts. Furthermore, we used structured questionnaires to measure public and stakeholder support before, during, and after the translocations. This resulted in a comprehensive and holistic understanding of the translocation effort that transcended jurisdictional boundaries and engaged stakeholders in meaningful and sustainable conservation of a large carnivore in a human‐dominated landscape.

## METHODS

### Lynx GPS tracking, translocations, integration, and interspecific interactions

**FIGURE 1:**
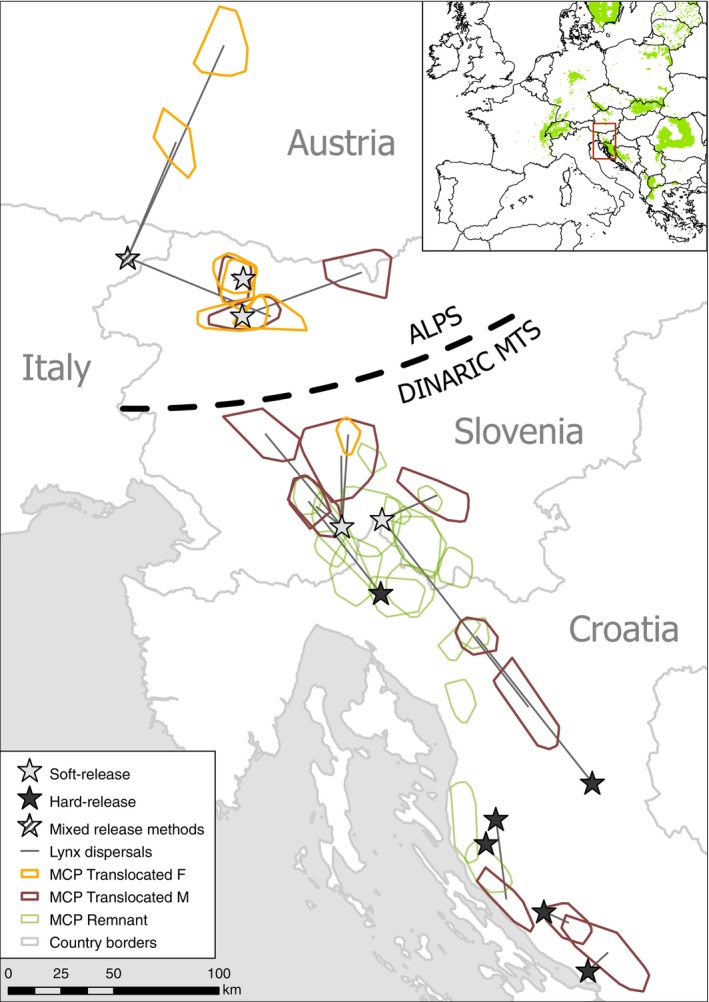
Map depicting the Dinaric and Alpine study areas with the release sites (stars), home ranges of the established translocated lynx (orange = females, brown = males) and GPS‐collared lynx from the remnant population (green). MCP stands for 95% minimal convex polygon. Straight black lines connect release sites with home‐range centroids of the established translocated lynx that dispersed after release. Note that only a small proportion of the remnant lynx population was collared. Not shown on the map are the GPS‐tracked F1 lynx and the translocated lynx that did not establish home ranges. The insert map shows location of the study area in Continental Europe with lynx distribution in 2016. *Source*: www.lcie.org.

We equipped 50 lynx with GPS‐GSM/Iridium‐VHF telemetry collars with a timer‐controlled drop‐off system (Vectronic Aerospace GmbH, Germany and Followit AB, Sweden) in the northern Dinaric Mountains and the Southeastern Alps in Italy, Slovenia, and Croatia (44°–46° N, 13°–16° E; Figure 1). These included all translocated lynx (*n* = 22; 2019–2024), some of their offspring (i.e., first‐generation offspring, hereafter “F1,” with at least one of the parents being a translocated lynx; *n* = 10; 2020–2024), and some of the lynx from the remnant population (*n* = 18; 2006–2024). The GPS fix schedule varied between individuals, seasons, and research focus (mean: 3 fixes per day; range: 0.5–96 fixes per day; for details, see external dataset Dataset1 available on Zenodo in Krofel et al., 2025, hereafter “Dataset1”). Some of the lynx (*n* = 7) were re‐captured for collar replacement in order to prolong the tracking period. Capturing and immobilization were done using standard protocols (for details, see Krofel et al., 2013). GPS‐tracking periods among individual lynx ranged between 1 and 1444 days (Dataset1).

All translocated lynx were first quarantined in the country where they were captured (minimum 3 weeks in Romania and Slovakia and minimum 1 week in Switzerland). After quarantining, the lynx were transported and hard released (i.e., without an acclimation period) in Croatia and Italy (*n* = 8) or soft released with an acclimation period in the enclosures built in the natural habitat at the release site in Slovenia and Italy (*n* = 14) (see Appendix S1: Section 1.1 and Dataset1 for details on capturing, quarantine, and soft releases). We considered translocated lynx as established when exhibiting a polygonal movement (Topličanec et al., 2022). If they dispersed <5 km (i.e., approximate radius of female home range) from the release site, we considered them as established in the release area. We considered lynx as integrated in the population if they established a permanent home range overlapping with conspecifics of the opposite sex for >1 year and/or were confirmed to have reproduced at least once.

To understand the integration of the translocated lynx into local ecosystems, we focused on two interspecific interactions that were reported as the most important for lynx ecology in the Dinaric Mountains, that is, predation and kleptoparasitism (Krofel, 2012). We compared predation parameters (feeding time and inter‐kill intervals) of the translocated individuals (*N* = 20) and lynx from the remnant population (*n* = 7) that had suitable GPS fix schedules to predict kill sites following the methodology described in Oliveira et al. (2023, 2025). Kills were identified with GPS location cluster (GLC) analysis with cluster criteria of 200‐m spatial buffer, 2 days of spatial window, and a minimum number of two GPS fixes (Oliveira et al., 2023, 2025). We visited a subset of the clusters in the field (*n* = 253, approximately 25% of all identified clusters) to determine prey composition (Krofel et al., 2014) and measured the time needed for the released lynx to make the first kill (Topličanec et al., 2022). When deciding which clusters to visit, we gave priority to fresh kills (i.e., shorter time since the predicted killing event) and to individuals for which less kill data were available at a given moment. We deployed camera traps on fresh kills (*n* = 62) to monitor prey consumption and detect scavengers benefiting from lynx kills (Krofel et al., 2019). Especially, kleptoparasitism by brown bears (*Ursus arctos*) was observed to have an important impact on lynx energetic input in the Dinaric Mountains, where bear densities are much higher than in the Alps (Krofel & Jerina, 2016). Therefore, we compared bear kleptoparasitism rates (i.e., proportion of lynx kills found by bears) and lynx feeding time (i.e., time lynx spent feeding on a given prey before abandoning it) for kills of translocated lynx in the Dinaric Mountains and the Alps (Krofel et al., 2012; Oliveira et al., 2025).

### Survival and mortality

We categorized lynx status at the end of GPS‐tracking periods following Andren et al. (2006) and Premier et al. (2025) as alive, disappeared, suspected illegal killing, confirmed illegal killing, roadkill, and natural mortality (definitions are provided in Appendix S1: Section 1.1). Suspected illegal killing, confirmed illegal killing, and roadkills were considered human‐caused mortality. Any potential mortality was immediately investigated when collars emitted a mortality mode or lynx were immobile for several days. All dead lynx that were found were necropsied.

We estimated survivorship based on lynx status at the end of each GPS‐tracking period (Dataset1). We excluded data for five lynx with unknown status (category: disappeared). One lynx from the remnant population was recaptured 10 years after his first capture, and thus, we considered the two tracking periods independently. We considered 45 tracking periods to estimate survival rates (Dataset1). We used the product‐limit (i.e., Kaplan–Meier) estimator (Kaplan & Meier, 1958) applied using the R package “survival” (Therneau et al., 2021) to determine survival rates for the three lynx groups (i.e., translocated lynx, F1 lynx, and lynx from the remnant population; Appendix S1: Figure S2).

### Reproduction

Reproduction of translocated females was detected by visiting den sites identified through GPS cluster analysis (Krofel et al., 2013) or with camera trapping (see *Population density and abundance*). Reproduction of translocated males was confirmed by genetic analyses of parenthood of the sampled kittens (*n* = 7; see *Genetic monitoring and inbreeding assessment*) or assumed when the presence of a family group (i.e., female with dependent kittens) was detected with camera traps inside established male home ranges (*n* = 15).

Litter sizes of translocated lynx and lynx from the remnant population were estimated using camera‐trap images of family groups detected between August and April. We used the largest number of kittens observed for a given female and season for further analysis. We used the Wilcoxon rank‐sum test to compare litter sizes between translocated lynx and lynx from the remnant population.

### Population density and abundance

We used camera‐trapping and spatial capture–recapture (SCR) analysis to assess changes in the density and abundance of the Dinaric lynx subpopulation in Slovenia and Croatia during the four consecutive years of the reinforcement (2019–2023), following methodology described in Fležar et al. (2023, 2025) (see also Appendix S1: Section 1.3 for further details). We classified the camera‐trapping sites (hereafter “location type”) based on their main characteristics: (1) lynx scent‐marking sites; (2) forest roads; and (3) other sites (Appendix S1: Section 1.3 and Figure S4). We selected at least two camera‐trapping sites in a potential home range of a studied population (Royle et al., 2011), with a 95% average MCP home‐range size of adult female lynx 97 km^2^ (Dataset1). Although some camera traps were active throughout the calendar year, we limited the camera‐trapping data for SCR modeling to August 15–February 15 (Appendix S1: Table S4) to meet the demographic closure assumption. However, to calculate population turnover (Appendix S1: Figure S8), we used all available records of independent individual lynx.

Lynx population density, baseline detection rate, and spatial scale parameters were estimated with maximum likelihood SCR models (Royle et al., 2011) using R package “oSCR” (Sutherland et al., 2019). We ran multi‐session models with four sessions defined as respective survey years in the Dinaric Mountains. We tested the effect of the local behavioral response (“b”) (Fležar et al., 2023; Royle et al., 2011) and the additive effect of survey year and sex on baseline detection rate and the spatial scale parameter, as suggested by Goldberg et al. (2015). Sex was included as a binary covariate (female as reference category) and survey year as factorial covariate. We included the additive effect of location type as a categorical three‐level covariate with marking site as a reference category (Appendix S1: Table S6), following Fležar et al. (2023).

For each survey year, we defined the extent of the effective sampling area, that is, the “state space” with the buffer width of 15 km and the resolution of buffer cells 2.5 × 2.5 km (Appendix S1: Figure S4; Fležar et al., 2023; Royle et al., 2011). We ranked candidate models based on Akaike information criterion (AIC) and their predictive power (AIC weight). The model with the best fit and highest predictive power was used to calculate the density and abundance of the lynx in the Dinaric Mountains for each survey year.

Systematic camera‐trapping data were not available for the newly established stepping‐stone subpopulation in the Southeastern Alps; therefore, we are not providing density and abundance estimates for this region.

### Genetic monitoring and inbreeding assessment

We used a dedicated laboratory for DNA extraction and PCR setup from non‐invasive and historic genetic samples (i.e., the laboratory was fully separated from other downstream laboratories, and we implemented very strict contamination prevention protocols). For genotyping, we used a set of 19 microsatellite markers (marker list together with protocols is provided in Appendix S1: Section 1.4). Genetic diversity was measured as the observed heterozygosity (Ho) and Nei's unbiased expected heterozygosity (He) (Nei, 1978). We used a traveling window analysis (Pazhenkova et al., 2025; Sindičić et al., 2013) to explore the erosion of genetic diversity caused by genetic drift in the Dinaric population with 40 samples as the width of the window. All analyses were programmed in R, and genetic data were handled with the R package “adegenet” (Jombart et al., 2008).

F1 individuals were detected through the private alleles not found in the Dinaric population prior to the translocation (26 private alleles on 11 loci), and they were assigned to possible parents through simple exclusion.

We used Wright's hierarchical structuring of inbreeding (Wright, 1931) to estimate the dynamics of inbreeding of the Dinaric population relative to the source population from the Slovakian Carpathians. We used the term “effective inbreeding” (Fe) (Frankham et al., 2010), where $\mathrm{Fe}=1-H_{\mathrm{Din}}/H_{\mathrm{SK}}$, with *H*_Din_ being heterozygosity in the Dinaric lynx and *H*_SK_ being heterozygosity in the source population in Slovakia (estimated to He = 0.592, using the same markers and 60 individuals) (Pazhenkova et al., 2025). We estimated the expected inbreeding depression δ using 12 diploid lethal equivalents (2B) (O'Grady et al., 2006). Remaining relative fitness compared to Slovak lynx was calculated as 1−δ (for details, see Appendix S1: Section 2.3).

To understand the effect of translocations, we explored three scenarios of inbreeding development after the reinforcement: excluding the effect of translocations (only lynx from the remnant population), including only lynx translocated to the Dinaric Mountains and their offspring (isolated stepping‐stone scenario), and including all translocated animals assuming future merging of the Alpine stepping stone with the Dinaric subpopulation (fully connected stepping‐stone scenario).

### Public and stakeholder attitude surveys

We measured support for lynx conservation and translocations among the general public and hunters through quantitative public attitude surveys, following the same methodological framework outlined in Majić et al. (2011). Surveys were based on a structured questionnaire consisting of 48 questions covering the following topics: general sentiment toward lynx, perceptions about lynx, knowledge and beliefs about lynx, opinions on various management measures and approaches, evaluation of information sources about lynx, demographic characteristics of the respondents, and the visibility of the LIFE Lynx project (Appendix S1: Section 1.5.2).

Surveys were conducted at the three key time points: start of translocations (2019), the midway point (2021), and the conclusion (2023). The goal was to ensure that the samples were as representative of the population as possible. To account for demographic changes, such as young individuals entering the sampling frame, immigration, emigration, and deaths, a new sample was generated for each survey iteration. There was no effort to sample the same people in consecutive surveys.

Random samples of adult (>18 years old) residents of the project area were obtained either through panel samples and implemented online (Croatia, Italy) or through sampling from the register of residents and implemented via regular mail (Slovenia, Croatia). For the latter, questionnaires were sent to the potential respondents and an envelope with prepaid return postage was included. In Slovenia, to improve the response rate among the initial non‐respondents, an additional reminder/thank you card was sent 7 days later. However, actual non‐respondents were not contacted further to explore reasons beyond non‐response.

Sample of hunters was obtained using following approaches: in Slovenia, 3–5 questionnaires were distributed to each local hunting organization within the project area; in Croatia, a computer‐assisted telephone interviewing (CATI) method was carried out and supplemented by an online survey shared through various social networks and portals relevant to the release areas; in Italy, a panel survey was carried out, and to increase the sample size, hunters themselves distributed additional copies of the questionnaire among their peers.

All the data were entered into an Excel spreadsheet. To ensure accuracy, a random sample of 3% of manually entered questionnaires was re‐checked for typing errors, with no mistakes detected. Further details on the survey methodology are provided in Appendix S1: Section 1.5.

## RESULTS

### Translocations, post‐release movement, integration, and interspecific interactions

We translocated 22 lynx (7 F, 15 M) from Romania (2 F, 10 M), Slovakia (3 F, 5 M), and Switzerland (2 F). These lynx were released to reinforce the remnant lynx population in the Dinaric Mountains in Slovenia (1 F, 5 M) and Croatia (6 M) and to create a new stepping‐stone subpopulation in the Southeastern Alps in Slovenia (3 F, 3 M) and Italy (3 F, 1 M) (Figure 1, Dataset1). All releases were supported and partly conducted by hunters from local hunting organizations or protected area managers (see also *Maintaining public and hunter support*).

Of the 22 translocated lynx, we considered 15 (68%) to have been successfully integrated into the population, 6 (27%) died or disappeared before reproducing, and the status of the remaining one animal is unknown (still in dispersal phase) (Dataset1). Among the soft‐released (*n* = 14) and hard‐released animals (*n* = 8) (Appendix S1: Figure S1), integration was successful for 71% and 63% of the lynx, respectively. Soft releases of lynx in the Slovenian Dinaric Mountains and Alps had the greatest population integration success (83% in both areas; Dataset1). Overall, 32% of the translocated lynx established permanent home ranges in the release area (<5 km from the release site), including 43% of the soft‐released and 13% of the hard‐released lynx. We observed the lowest dispersal rates of lynx released in the Slovenian Alps, where 83% established permanent home ranges in the release area (Figure 1, Dataset1). One of the males released in the Slovenian Dinaric Mountains moved to the Alps and back, indicating opportunity for functional connectivity between the Alpine stepping‐stone and Dinaric subpopulations. The median (± interquartile range) distance between the release site and centroid of established home ranges was 23.20 ± 34.97 km (*n* = 19; mean = 27.91 km). The median distances were 41% shorter for the soft‐released lynx (19.57 ± 37.58 km; *n* = 13) than for the hard‐released lynx (32.92 ± 33.96 km; *n* = 6), although the difference was not statistically significant (Kruskal–Wallis χ^2^ = 0.77, *p* = 0.38).

On average (±SD) translocated lynx made the first large kill 8.75 ± 6.85 days after release (*n* = 12; Dataset1). The mean inter‐kill intervals (±SE) for large prey of the translocated lynx (4.37 ± 0.12 days; *n* = 621 predicted kills) were almost identical to those from the remnant population (4.38 ± 0.14 days; *n* = 385; Kruskal–Wallis χ^2^, *p* = 0.88) (Dataset1). Translocated lynx mainly (83%) killed roe deer (*Capreolus capreolus*), and we observed 19 species of scavengers feeding on their kills (see Appendix S1: Tables S1 and S2 for full lists of prey and scavenger species). Brown bear kleptoparasitism rates were considerably lower in the Alps (4.0%) than in the Dinaric Mountains (32.3%), where bear densities are much higher. Accordingly, we observed 15% longer feeding times of the translocated lynx in the Alps (mean ± SD: 2.41 ± 0.08 days) than in the Dinaric Mountains (2.09 ± 0.07; Kruskal–Wallis χ^2^, *p* = 0.006).

### Survival and mortality causes

**FIGURE 2:**
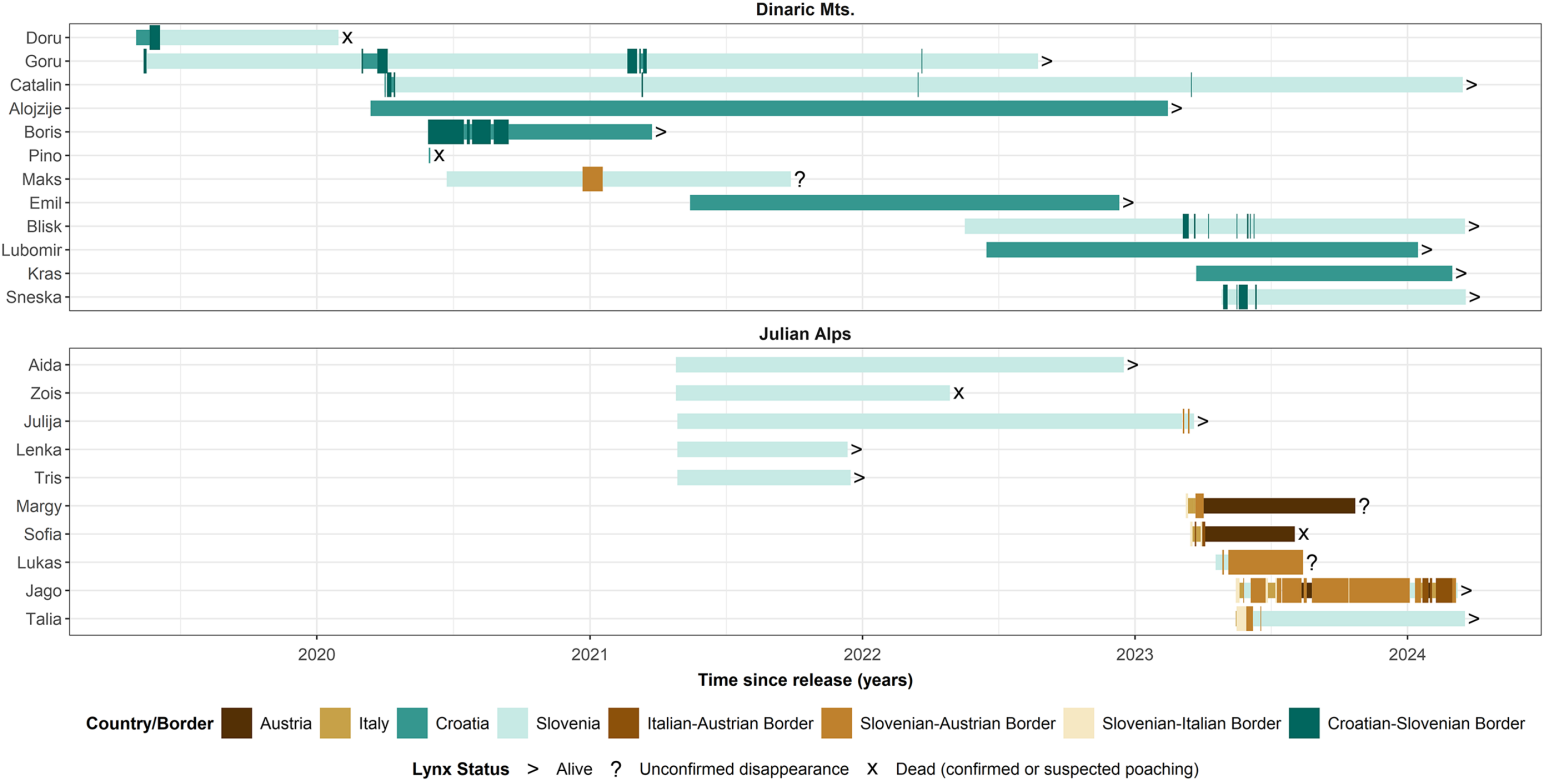
Spatiotemporal representation of lynx movements within GPS‐tracking periods and status at the end of tracking periods for 22 lynx translocated to the Dinaric Mountains (above) and the Julian Alps (below). Colors and width of lines indicate location at given countries or border zones (i.e., at 5‐km buffer around the border).

Among the 50 GPS‐tracked lynx, 33 survived the entire GPS‐tracking period, 5 disappeared, and 11 were confirmed or suspected to die (mean length of the tracking periods: 380 days, range: 1–1444 days; Dataset1). We did not find significant differences in the survival among the three considered groups (i.e., offspring of translocated lynx, F1, *n* = 10; translocated individuals, *n* = 22; lynx from the remnant population, *n* = 18) as there was high variation in the survival rate (i.e., large CIs) within each group (Kaplan–Meier, *p* = 0.52; Appendix S1: Figure S2). Confirmed or suspected mortality was the highest for the lynx from the remnant population (33%), followed by the translocated (18%) and F1 lynx (11%) (Dataset1). All confirmed or suspected mortalities among the translocated and F1 lynx (*n* = 5) appear to be human caused (confirmed or suspected illegal killing). Persistence seemed particularly low for the four translocated lynx that dispersed into Austria or to the region along the Austrian border (Figure 2). Among the six lynx from the remnant population that died, two were probably human caused (roadkill and suspected illegal killing) and four were natural. Among natural mortalities, we recorded two cases of heart failure connected with congenital atrial septal defects, one case of pneumonia, and in one case, the exact cause was unclear. Moreover, we detected additional morphological deformations or their manifestations possibly linked to inbreeding among the lynx from the remnant population (heart murmurs, skeletal deformations, and double ear tufts on the same ear instead of normal, single ear tuft; Appendix S1: Figure S3). No deformations or natural mortalities were observed among the translocated or F1 lynx.

### Reproduction

Among the 22 translocated lynx, 13 (59%) reproduced by 2024 and 3 more individuals have the potential for future reproduction (i.e., they are still alive, but we did not yet confirm their reproduction; Dataset1). We were able to confirm the parentage of translocated lynx in 10 out of 23 litters detected by camera trapping. We recorded 54 kittens with presumed or confirmed parentage from the translocated lynx. This is likely an underestimate because males may have sired additional offspring during extra‐territorial mating excursions. Such excursions were recorded in 10 out of the 15 mating seasons of the 9 GPS‐tracked translocated males. Also not included in the reported number of kittens is the reproduction of F1 lynx (two litters confirmed so far).

We obtained data from 97 lynx litters by camera trapping. When at least one of the parents was presumed or confirmed to be a translocated or F1 lynx, litter sizes were 37% larger compared to the litters with both parents from the remnant population (F1 litters: 2.21 ± 0.78 kittens, *n* = 24; only confirmed F1 litters: 2.30 ± 0.95 kittens, *n* = 10; remnant litters: 1.62 ± 0.68 kittens, *n* = 73; *p* < 0.001).

### Changes in population density and abundance

We deployed camera traps in the Dinaric Mountains at 225–329 sites per survey year in 2019–2023, resulting in 116,902 camera‐trapping days. For SCR modeling, we used lynx data from 1021 independent occasions to create 234 individual capture histories (Appendix S1: Table S5). The model with the most support included survey year, local behavioral response, sex, and location type as effects on the baseline detection probability parameter and sex as an effect on the spatial scale parameter (Appendix S1: Table S3). Males were detected roughly twice as often as the females, and the camera traps deployed at marking sites had approximately two and three times higher probability of detecting lynx than the camera traps deployed on roads and other locations, respectively (Appendix S1: Table S7).

**FIGURE 3:**
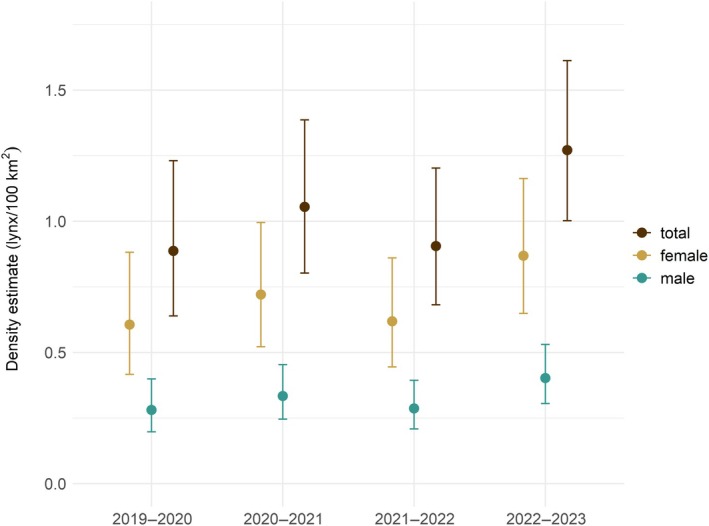
Changes in density estimates for the lynx in the Dinaric Mountains during the reinforcement (2019–2023). Estimates (with 95% CIs) are shown for each survey year for females, males, and all independent lynx.

During the 4‐year reinforcement efforts in the Dinaric Mountains, the mean lynx population density increased by 44.3% (from 0.88 [95% CI: 0.63–1.23] independent lynx/100 km^2^ in 2019–2020 to 1.27 [1.00–1.61] in 2022–2023), with the highest increase in the last survey year (Figure 3, Appendix S1: Table S3). This increase occurred without any major changes in the effective area surveyed, which ranged between 12,206 and 12,350 km^2^ per survey year (Appendix S1: Figure S4, Table S5). The estimated densities correspond to a 42% increase in abundance from 110 (95% CI: 79–152) adult lynx in 2019–2020 to 156 (123–198) adult lynx in 2022–2023 in the state space (Appendix S1: Table S5).

Despite the population growth, we observed a relatively high turnover rate (i.e., compared to the annual survival rates of 70%–95% for lynx populations across Europe; Premier et al., 2025) in the population, which remained similar throughout the survey period (34%–36% of individually identified lynx were detected in the following year; Appendix S1: Figure S8).

### Impact on genetic diversity and inbreeding

We analyzed 750 genetic samples from the Dinaric Mountains and the Southeastern Alps collected in the 2010–2023 period, including the translocated lynx, and 204 historical genetic samples from 1979 to 2010 (Appendix S1: Section 2.3). We identified 238 individuals and used genotypes of 228 individuals (1979–2010 = 88, 2011–2016 = 25, 2017–2023 = 117) for downstream analyses. Some animals were excluded because of incomplete field data (*N* = 3), and we excluded the translocated animals that we know had died before reproduction or had no chance to reproduce because they dispersed outside of the species range, where no potential mates were available (*n* = 7).

**FIGURE 4:**
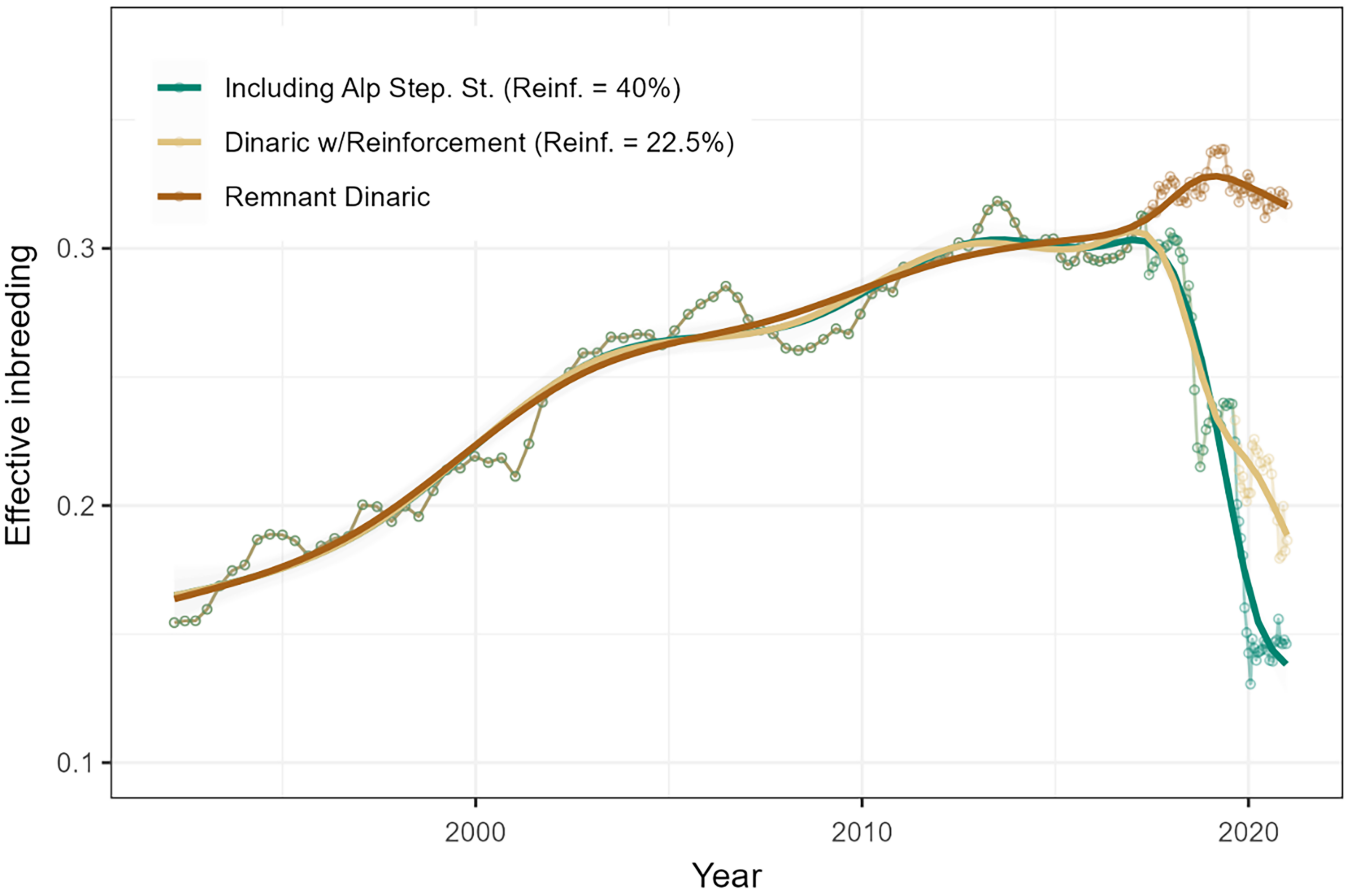
Effective inbreeding (Fe) of Dinaric lynx relative to the population in the Slovak Carpathians (source for the remnant population from the 1973 reintroduction), calculated using a 40‐sample traveling window. Remnant Dinaric: calculated without the translocated lynx and their offspring, exploring the situation without the effect of translocations; Dinaric w/Reinforcement: including lynx translocated to the Dinaric Mountains and their offspring; Including Alp Step. St.: with all translocated lynx, including the Alpine stepping‐stone subpopulation. The “Reinf.” value indicates the proportion of translocated animals and their offspring in the final traveling window (right end of the graph).

Without the translocated lynx, inbreeding would have reached 0.32 at the end of the project (2024), with expected inbreeding depression of δ = 0.85 (i.e., the fitness of lynx from the Dinaric remnant population is expected to be 15% of those in the source population). It is difficult to precisely estimate the effect of the reinforcement as it will take several generations for allelic frequencies to stabilize and the population to get into the Hardy–Weinberg equilibrium (Cornuet & Luikart, 1996), but we can already see that this effect is considerable. Even assuming no connectivity with the Alpine stepping‐stone subpopulation, inbreeding in the Dinaric lynx would be around 0.19 and inbreeding depression δ = 0.68 at the end of the translocation efforts, suggesting that fitness already more than doubled due to the reinforcement. If we assume full connectivity with the Alpine stepping‐stone subpopulation, inbreeding would drop to 0.08 when translocated animals and their offspring form around 40% of the population, with expected inbreeding depression dropping to δ = 0.39 (Figure 4).

### Maintaining public and hunter support

In the anticipation of the reinforcement project, considerable effort was directed toward stakeholder engagement and securing public support for the translocation initiative, which continued throughout the translocation period. These efforts included the development of local consultative groups as platforms for communication and partnership with local communities (for details, see Appendix S1: Section 1.5.1), working with local school teachers, designing tourism initiatives with local providers, ongoing engagement with the local, national, and international media, and upheld transparent communication with the wider public through various channels, such as working with media and celebrity ambassadors, organizing public lectures and other events, maintaining an informative website (https://www.lifelynx.eu) and social media pages, as well as producing targeted publications, documentaries, and books.

Special attention was given to engaging and maintaining transparent communication with hunters, who were identified as the key stakeholders for lynx conservation in the region. This included regular communication, organized presentations, and discussions with local hunting clubs, and one‐on‐one meetings during joint efforts in the field. The latter mostly took place in the frame of lynx camera‐trapping monitoring and GPS tracking of the collared lynx. Over 200 hunters participated in camera trapping in Slovenia, Croatia, and Italy. To ensure continuous feedback, hunters were regularly updated on monitoring results and other project activities through publications in hunting magazines, social media, lectures at hunting events, and frequent personal communication with each collaborating hunter. Over the years of this partnership, we have established strong trust‐based relationships with hunters across most of the lynx distribution in the Dinaric Mountains and the Julian Alps and ensured that hunters maintained a proactive role in lynx research and conservation.

Hunters also played a crucial role in the translocation and releasing of lynx, as all release locations were decided in collaboration with local hunters and protected area managers, who were also responsible for building the soft‐release enclosures, as well as for feeding and providing security for the lynx while they were kept in the soft‐release enclosures. Furthermore, the hunting organization (Hunters Association of Slovenia) led the education of law enforcement to prevent the illegal killing of lynx and other wildlife (for details, see Appendix S1: Section 1.5.1). To further enhance hunter stewardship for lynx conservation, we regularly highlighted the role played by hunters in the efforts to restore the lynx population when communicating about project activities to the general public (Appendix S1: Section 1.5.1).

**FIGURE 5:**
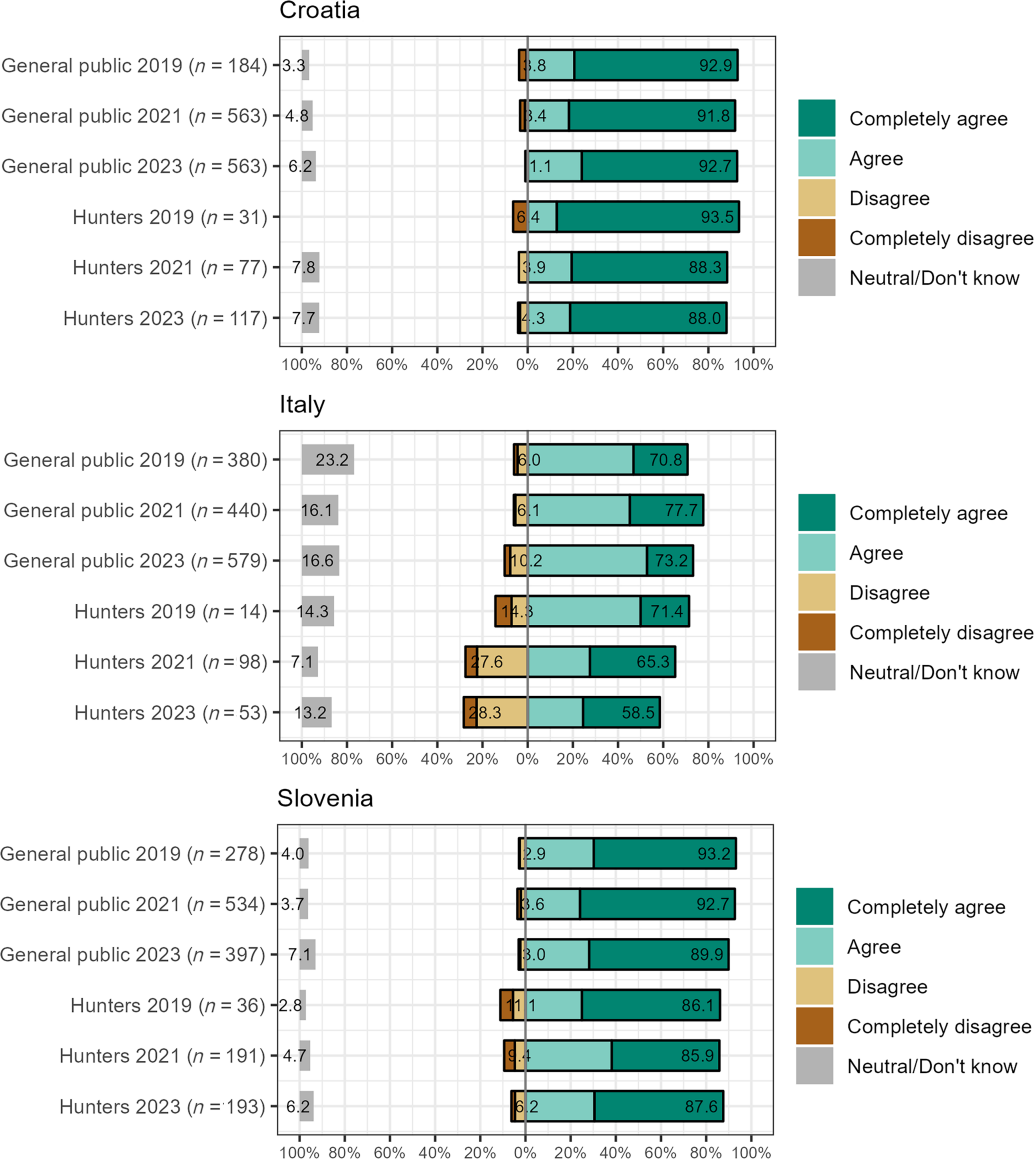
Responses to the statement: “It is important to maintain lynx in Croatia/Italy/Slovenia for the future generations.” Respondents answered for their respective countries.

Results of the attitude surveys indicate that general public and hunter support for lynx conservation remained consistently high throughout the translocation period. Respondents' commitment to lynx conservation, as indicated by their bequest value orientation (i.e., value that individuals place on the ability to pass a resource to the future generations), remained high among the general public and hunters until the final year (2023), with the highest levels of support observed among the Croatian and Slovenian respondents (Figure 5). Additionally, support for lynx translocations was initially high and increased during the mid‐project period. As the number of lynx released increased toward the end of the project, there was a slight decline in support among the Italian and Croatian respondents, although the majority still expressed support for the translocation efforts (Appendix S1: Figure S10; entire dataset is available as Dataset2 on Zenodo in Krofel et al., 2025).

## DISCUSSION

Rescue of the Dinaric lynx population demonstrates the feasibility of using translocations to recover a highly inbred large carnivore in a human‐dominated landscape and across national borders. Besides carefully designed translocations, the efforts to communicate regularly and transparently with the public and engage key stakeholders in the project implementation helped make the effort a success. Our study also provides a rare example of a comprehensive monitoring program implemented on a transboundary level during a translocation effort. We monitored both biological and social parameters before, during, and at the end of translocation, thus ensuring that we could evaluate and adapt management options throughout the process and ensure a holistic understanding of the implemented reinforcement and reintroduction. We suggest that this project may serve as a blueprint for planning future efforts to re‐establish healthy populations of large carnivores in human‐dominated landscapes.

### Mortality, reproduction, and integration success

Lynx that were soft‐released had considerably lower probabilities of leaving the release sites and showed somewhat higher success rates of integration into the remnant population than hard‐released lynx. This is in line with previous research on translocations of carnivores and other terrestrial animals (Resende et al., 2021; Thomas et al., 2023). However, it also appeared that the presence of conspecifics of the opposite and same sex played an important role in post‐release behavior. This could also explain differences in the dispersal rates between soft‐released animals in the Slovenian Alps compared to the Dinaric Mountains and warrants further analysis.

Natural mortality causes were dominant among the remnant animals that were found dead, and especially congenital heart defects with other morphological deformations indicate possible effects of inbreeding depression. Mortality was lower among translocated lynx, where the main cause was illegal killing, similar to other lynx populations in Europe (Premier et al., 2025). An especially important concern is the rapid disappearance of lynx that moved across or to the border with Austria, which aligns with high illegal killing rates of lynx and other large carnivores reported for this region (Heurich et al., 2018; Kaczensky et al., 2011; Molinari et al., 2021). To address the threat of illegal killing, the LIFE Lynx project invested considerable efforts in partnership with hunters in Slovenia, Croatia, and Italy (for details, see Appendix S1: Section 1.5), including establishing the first specialized anti‐poaching police team in the region.

Six months after releases, the survivorship of translocated lynx (86%) was higher than the average survivorship (66%) reported by Thomas et al. (2023) for recent large carnivore translocations. The majority of translocated lynx also succeeded in reproducing, and we observed significantly higher reproductive success compared to the lynx from the remnant population, probably related to lower inbreeding levels. An important additional factor that likely contributed to high rates of survivorship was that we used only wild‐born individuals who had innate hunting ability. This is supported by no signs of reduced kill rates and the relatively short periods needed to capture the first large prey after release. Likely, this prevented starvation, which is often a leading cause of mortality among translocated predators (Devineau et al., 2010). Bear kleptoparasitism rates for translocated lynx in the Dinaric Mountains were similar to the lynx from the remnant population (Krofel & Jerina, 2016), but they were considerably lower among the lynx translocated to the Alps, where bear densities are low. This could explain longer feeding times (and therefore higher food intake) in the Alps, which could, from this perspective, represent a more advantageous lynx habitat. Overall, the observed interspecific interactions involving translocated animals were similar to the lynx from the remnant population (Krofel et al., 2012, 2014, 2019), which suggests that most of the translocated lynx became quickly integrated into the local ecosystems.

### Impact on population demography

After the 2000s, the Dinaric lynx population experienced a drastic decline and became functionally extinct in the Alpine study area (Fležar et al., 2021). After the reinforcement was initiated in 2019, the population trend in the Dinaric Mountains reversed and the lynx abundance increased by more than 40% in the following 3 years. The increase was mainly connected with increasing density and was non‐linear in time, with a major change detected in the last survey year. This time lag was likely due to the gradual integration of new animals in the population and the time needed for their offspring (>50 kittens detected) to mature to independent animals, which are considered in the camera‐trapping monitoring. Current population densities already surpass many of the density estimates from other reintroduced lynx populations in Europe and approach those of the source population (reviewed in Fležar et al., 2023). With saturation of the core area, we expect that in the following years, the population will start expanding to the adjacent areas with suitable habitat.

Measuring a large‐scale demographic impact of translocations is still rare (Taylor et al., 2017) mainly due to considerable logistic constraints and effort required to conduct transboundary monitoring over vast ranges occupied by large carnivore populations (Fležar et al., 2023; Tourani, 2022). Our study demonstrates that citizen science involving close partnership with local amateur and professional hunting organizations and protected area managers can be an efficient approach for obtaining multi‐year datasets for a robust population density assessment of an elusive large carnivore. This approach requires considerable effort in communication and coordination, but at the same time, it results in increased trust in monitoring results and increased support for conservation measures among the involved stakeholders. Furthermore, in combination with intensive international collaboration, such camera‐trapping surveys can become an achievable goal even over large (>10,000 km^2^) scales and spanning across multiple countries and management jurisdictions, which can otherwise be a major limitation for carnivore translocations (Port et al., 2024).

A priority for the future is to expand this monitoring to the southern end of the Dinaric population in Bosnia and Herzegovina, where very limited information about lynx is currently available.

### Impact on genetic diversity and inbreeding

The genetic erosion of the Dinaric lynx population since the 1973 reintroduction and critical levels of inbreeding (Fe > 0.3) resulted in a severe inbreeding depression (expected δ = 0.85), suggested also by the observed congenital heart defects and morphological deformations. The post‐reinforcement situation shows a dramatic improvement, with a considerably increased expected heterozygosity and the related expected fitness more than doubling since the reinforcement. This is corroborated by other field data showing encouraging signs of recovery (increased population size, larger litter sizes, and decreased natural mortality). We can expect that inbreeding in the population will continue dropping as offspring of the translocated animals spread through the population and allelic frequencies stabilize (Cornuet & Luikart, 1996).

Over the next decade, the status of the population will depend on the continued reproductive performance of the translocated animals and their offspring. In the well‐studied case of the reinforcement of the Florida panther (*Puma concolor coryi*) population, considerable heterosis (fitness advantage of outbred animals) was observed in the F1 animals, which contributed to the rapid expansion of the introduced genes in the population (Johnson et al., 2010). After the reinforcement, panther numbers increased threefold, heterozygosity doubled, survival and fitness measures improved, and inbreeding correlates declined significantly. We have already started observing similar changes in the Dinaric lynx. Moreover, as most of the translocated animals originated from another part of the Carpathian lynx range (Romania) than the remnant population (Slovakia), the resulting F1 offspring are even more outbred than the lynx in the source populations. A continuous monitoring program should be a priority to keep track of future developments, especially since the population remains small, isolated, and bottlenecked. In the long term, this will require creating a large Central European metapopulation or conducting further periodic translocation of outbred lynx (Pazhenkova et al., 2025).

### Maintaining public support

Any predator translocation or increase in carnivore populations can result in intensified public concerns (Treves & Karanth, 2003; Wilson, 2018). Thus, the gradual erosion of public support and opposition from key stakeholders (hunters) were among the main concerns in the planning phase of lynx translocation. Various stakeholder engagement efforts along with intensive communication campaigns within the LIFE Lynx project likely contributed to sustaining high support for lynx conservation among the local hunters and general public throughout the translocation efforts and population increase. Although the majority was also in favor of the translocations throughout the process, this support has slightly tapered off toward the end of the project in Italy and Croatia, possibly reflecting the lower perceived need for further translocations, when the population was no longer in immediate risk of extinction. With the lynx population steadily recovering, future stakeholder engagement strategies should pivot toward (1) addressing stakeholder‐specific and country‐specific concerns regarding the implications of the lynx recovery and (2) effectively communicating the necessity of long‐term measures to ensure the establishment of a viable lynx metapopulation at a transnational level.

### Future perspectives

Central Europe is a highly fragmented landscape, and none of the existing forest complexes are large enough to host a viable isolated lynx population. Therefore, the establishment of a functional transboundary metapopulation is needed to mitigate the negative effects of habitat fragmentation and ensure long‐term viability (Mueller et al., 2022). Genetic restoration of the Dinaric population and the creation of a new stepping‐stone subpopulation in the Julian Alps reported in this study represent a major advance toward reaching this long‐term vision. The next step is the creation of additional stepping stones that could eventually re‐establish connectivity across the Alpine arc with (Western) Alpine and Jura populations in Switzerland and France (Molinari et al., 2021). However, the high illegal killing rates in Austria and neighboring regions in the Alps will likely be a major obstacle in achieving this goal. This calls for a targeted, multi‐sectoral approach, which considers the practice of stakeholder engagement reported here and acknowledges the given cultural context. Further efforts, potentially through the creation of new stepping stones, should be dedicated also toward establishing connection with the critically endangered and inbred Balkan lynx population toward the southeast (Melovski et al., 2022).

As UN decade on ecosystem restoration progresses, numerous carnivore restoration projects are currently being planned or already initiated globally, including efforts to return lynx to Great Britain and several parts of Germany, gray wolves (*Canis lupus*) to Colorado, USA, cheetahs (*Acinonyx jubatus*) to India, and leopards (*Panthera pardus*) to Saudi Arabia. Lessons learned from our comprehensive conservation endeavors coupled with the multidisciplinary monitoring of biological and social factors could provide an important example to facilitate these carnivore restoration efforts.

## AUTHOR CONTRIBUTIONS

**Miha Krofel:** Conceptualization, formal analysis, funding acquisition, investigation, project administration, supervision, visualization, writing—original draft, writing—review and editing. **Urša Fležar:** Data curation, formal analysis, investigation, methodology, project administration, visualization, writing—original draft, writing—review and editing. **Rok Černe:** Funding acquisition; investigation; project administration; supervision; writing—review and editing. **Lan Hočevar:** Data curation; formal analysis; investigation; writing—original draft; writing—review and editing. **Marjeta Konec:** Data curation; formal analysis; investigation; project administration; writing—original draft; writing—review and editing. **Aleksandra Majić Skrbinšek:** Data curation; formal analysis; funding acquisition; investigation; project administration; supervision; visualization; writing—original draft; writing—review and editing. **Tomaž Skrbinšek:** Data curation; formal analysis; funding acquisition; investigation; methodology; supervision; visualization; writing—original draft; writing—review and editing. **Seth Wilson:** Writing—original draft; writing—review and editing. **Bernarda Bele:** Investigation; writing—review and editing. **Jaka Črtalič:** Investigation; writing—review and editing. **Tomislav Gomerčić:** Investigation; writing—review and editing. **Tilen Hvala:** Investigation; writing—review and editing. **Jakub Kubala:** Investigation; writing—review and editing. **Pavel Kvapil:** Investigation; writing—review and editing. **Meta Mavec:** Investigation; writing—review and editing. **Anja Molinari‐Jobin:** Funding acquisition; investigation; project administration; writing—review and editing. **Paolo Molinari:** Funding acquisition; investigation; writing—review and editing. **Elena Pazhenkova:** Investigation; writing—review and editing. **Hubert Potočnik:** Investigation; writing—review and editing. **Teodora Sin:** Investigation; writing—review and editing. **Magda Sindičić:** Investigation; project administration; supervision; writing—review and editing. **Ira Topličanec:** Investigation; writing—review and editing. **Teresa Oliveira:** Data curation; formal analysis; investigation; methodology; visualization; writing—original draft; writing—review and editing.

## CONFLICT OF INTEREST STATEMENT

The authors declare no conflicts of interest.

## Supporting information


Appendix S1.

## Data Availability

Data (Krofel et al., 2025) are available on Zenodo at https://doi.org/10.5281/zenodo.15252332.
