# Supplementary material for: Multidisciplinary monitoring and stakeholder engagement support large carnivore restoration in human‐dominated landscape
Source: Ecol Appl. 2025 May 26;35(3):e70052. doi: 10.1002/eap.70052 (PMC12104966; doi:10.1002/eap.70052)
Supplement: Supplementary file 1 — Appendix S1. [file EAP-35-e70052-s001.pdf]

Appendix S1
Supporting information for:

Multidisciplinary monitoring and stakeholder engagement support large carnivore restoration in human-dominated landscape

Ecological Applications

Miha Krofel, Urša Fležar, Rok Černe, Lan Hočevar, Marjeta Konec, Aleksandra Majić Skrbinšek, Tomaž Skrbinšek, Seth Wilson, Bernarda Bele, Jaka Črtalič, Tomislav Gomerčić, Tilen Hvala, Jakub Kubala, Pavel Kvapil, Meta Mavec, Anja Molinari-Jobin, Paolo Molinari, Elena Pazhenkova, Hubert Potočnik, Teodora Sin, Magda Sindičić, Ira Topličanec, Teresa Oliveira

Content

Section S1: Methods	pages 2 – 6
Section S2: Results	pages 7 – 8
Section S3: Figures S1-S10	pages 9 – 18
Section S4: Tables S1-S8	pages 18 – 26
Section S5: References	pages 27 – 29

Section S1: Methods

S1.1 Surveys in source populations, translocations, movements, survival and mortality

Before capturing lynx for translocation, source populations were surveyed to confirm that removal of animals needed for translocations would be sustainable and would not jeopardize these populations (for details see Kubala et al. 2018; Sin et al. 2021).

Translocated lynx were quarantined for a minimum of three weeks in Romania and Slovakia and for a minimum of one week in Switzerland. Protocols used for capturing, quarantine, transport and releases of lynx are available on the project webpage (https://www.lifelynx.eu/protocols).

Soft-release followed an acclimation period at the release site lasting on average 19.1 ± 14.7 days (range: 3-48 days) in Slovenia and Italy (for details see Dataset1 available via Zenodo at Krofel et al. 2025; hereafter 'Dataset1').

The process of integration of each translocated lynx was continuously monitored, which guided us in decisions on where and when to release the next animals. Relatedness with already released animals was also considered in the release decision-making.

Definitions of categories describing lynx status at the end of GPS-tracking periods:
- alive: lynx still tracked at the end of the study or moving when the collar drop-off was triggered
- disappeared: collar signal lost (i.e. no more GSM/Iridium data transmission and no VHF signal found in the lynx home range) before the expected battery lifetime, but no systematic monitoring with camera-trapping or snow-tracking was conducted in the area of disappearance to provide further information; lynx mostly likely disappeared because the collar failed or lynx was illegally killed, although it may be also possible that lynx died of natural causes in a place where GSM/Iridium and VHF collar signals could not be obtained
- suspected illegal killing: collar signal lost well before the expected battery lifetime and lynx disappeared from camera-trapping records at about the same time
- confirmed illegal killing: evidence of illegal killing found, e.g. shot lynx or destroyed collar
- road-kill: lynx killed in a vehicle collision
- natural mortality: dead lynx found without signs of human-caused mortality

Because most of the tracked lynx were adults at the time of the collaring/release (i.e., ≥2 years; 76%, n=36), and the collaring/release took place mostly during winter/spring (96%, n=43), we did not include age and season as covariates in our model of lynx survival. GPS-tracking periods that ended without mortality were right-censored, i.e. the survival time was not observed because the tracking ended before the animal died (collar removed, drop-off, or failure).

S1.2 Predation and kleptoparasitism

For each kill, we calculated the feeding time and the search time. We defined the feeding time as the period between when the prey was killed and when lynx abandoned the kill (Krofel et al. 2012; Oliveira et al. 2025). We used feeding time as a proxy of lynx food intake per kill. The search time was defined as the period between the abandonment of current prey item and the next kill. The inter-kill interval (analogous to kill rates) considers the time passed between the start of a given kill and the start of a previous kill, and includes the feeding time (capturing and consuming prey) and searching time (the time spent searching for prey) (Oliveira et al. 2023, 2025).

S1.3 Camera trapping

S1.3.1 Fieldwork

We followed the camera trapping guidelines (Stergar and Slijepčević 2017) for camera set up at three types of locations: lynx scent-marking sites (Allen et al. 2017), forest roads (i.e. unpaved forest roads and logging trails), and other sites (i.e. off-road sites where no indication of lynx scent marking was observed). Camera traps were set up exclusively in forested areas, which is the optimal habitat of lynx in the Dinaric

Mountains (Fležar et al. 2023). We used cameras with white flash (CuddeBack X-Change Color Model 1279, Cuddeback, Green Bay, Wisconsin; Reconyx HyperFire 2 Professional White Flash Camera HP2W, Reconyx, Inc., Holmen) or cameras with black (940 nm light wave) or regular infrared light (850 nm light wave; CuddeBack X-Change Color Model 1279, Cuddeback, Green Bay, Wisconsin; StealthCam STC-G42NG, Stealth Cam, Irving; Moultrie M40-i, PRADCO Outdoor brands, Birmingham; LTL Acorn models Ltl-6310WMG and Ltl-6511WMC; Browning Spec Ops Elite HP4, Browning Trail Cameras, Birmingham, Alabama).

In Slovenia, camera traps were set at all three types of locations, while camera traps were primarily set at marking sites in the Northern part of Croatia (Gorski Kotar), and at roads in the Southern part of Croatia (Velebit). We limited the camera trapping data for this survey to the sites located South of the A1 highway in Slovenia. The highway represents a serious barrier to the lynx home range, i.e. telemetry tracking showed no lynx established a territory overlapping the highway, although some dispersing lynx managed to cross it (Krofel et al. 2006, 2021). The data from the Alpine part was not considered for the SCR modeling, as the stepping stone subpopulation was not yet connected to the Dinaric population and only consisted of the known translocated lynx and their offspring at the time of this study. The mean trap spacing (Table S4) and spatial scale parameter calculated by the models (see Results) confirmed that designs in all regions and the entire study area fit the standard recommendation of 2σ (Dupont et al. 2021; Efford and Fewster 2013).

S1.3.2 Data preparation

Trained observers (n=9) annotated lynx records with sex, age (juvenile / independent) and individual identity, whenever possible, following the identification guidelines (Choo et al. 2020). Only data about individually-identified independent lynx originating from high-quality records where the coat pattern was clearly visible were used to build individual capture histories. International collaboration between Slovenia and Croatia allowed for uninterrupted exchange of data, including identifying lynx with cross-border territories. This was done also with the help of artificial-intelligence-based tools (www.whiskerbook.org). Records of lynx where identification was not possible were discarded (less than 20% of records each survey year), as well as records of juveniles (Andrén et al. 2006; Duľa et al. 2021). Lynx records and trap deployment data were exported directly from the Camelot software (Hendry and Mann 2017), which was used for camera trapping data annotation, and reorganized to fit SCR analysis.

S1.3.3 SCR modelling

Lynx density, baseline detection rate and spatial scale parameter were estimated with maximum likelihood spatial capture-recapture models (Royle et al. 2013) using oSCR package (Sutherland et al. 2019) in R software v. 4.1.0. We ran multi-session models with four sessions defined as respective survey years (2019-2020, 2020-2021, 2021-2022, 2022-2023) in the Dinaric Mountains (i.e. not including the Alpine part). At least 20 spatial recaptures were confirmed for each survey year and for the entire transboundary study area (Dinaric Mountains; Table S5) (Efford et al. 2004).

For each survey year, we defined the extent of the effective sampling area, i.e. the "state space" with the buffer width of 15 km and the resolution of buffer cells 2.5 x 2.5 km, following the recommendations of Royle et al. (2013) and our own findings (Fležar et al. 2023). We restricted the state space following the same criteria as described in Fležar et al. (2023; see also Figure S5). We ranked candidate models based on Akaike Information Criterion (AIC) with models having $\Delta AIC \leq 2$ considered having substantial support (Burnham and Anderson 2004), and their predictive power (AIC weight; Johnson and Omland 2004). Among the highest-ranking models, the one with the best fit (lowest AIC value) and the highest predictive power (highest AIC weight) was used to calculate the density and abundance of lynx in the Dinaric Mountains for each survey year.

S1.4 Genetics

Tissue samples from dead lynx were stored in 95% non-denatured ethanol and stored at -20°C. Blood was preserved on blood stain cards (Qiagen). These high-quality DNA samples were processed using a manual DNA extraction kit (Sigma GenElute Mammalian Genomic DNA Miniprep Kit) following the manufacturer's protocol.

Non-invasive genetic samples: Urine samples (collected in snow) were stored in DETs buffer, and hair samples were stored in sealed bags with desiccant (silica). Saliva samples were collected with forensic swabs that already have desiccant in the swab tube. Scats were collected in ethanol. Sampling material was prepared and distributed at the beginning of the project, instructions for collecting were presented in dedicated guidelines (Skrbinšek 2017). DNA in non-invasive, historic genetic and eDNA samples is of very low quality and quantity, and contamination (especially with PCR products) is a serious issue. We used a dedicated laboratory for samples storage, DNA extraction and PCR setup. For non-invasive samples we used MagMAX DNA Multi-sample Kit (Thermo Fisher Scientific). The extraction protocol is implemented on a liquid handling robot (Hamilton Starlet), samples IDs are read and handled through barcodes.

We used ten microsatellite markers for individual ID run in a single multiplex: Fca132, Fca201, Fca247, Fca293, Fca391, Fca424, Fca567, Fca650, Fca723, Fca82. The best (reference) sample of each detected animal was amplified using 9 additional markers (F115, F53, Fca001, Fca132, Fca161, Fca369, Fca559, Fca742, HDZ700 (Menotti-Raymond et al. 1999; Menotti-Raymond et al. 2005; Williamson et al. 2002), bringing the total number of studied microsatellites to 19. SRY locus was used to determine sex of the animal. Microsatellites were amplified in 3 multiplexes, using Platinum multiplex PCR Master Mix (ABI). Protocols from (Polanc et al. 2012) were adapted according to the Platinum kit user guide. Good quality tissue and blood samples were re-amplified twice. For non-invasive samples, we used a modified multiple-tube approach (Adams and Waits 2007; Taberlet et al. 1996) with up to 8 re-amplifications of each sample according to the sample's quality and matching with other samples. In the first screening process, each sample was amplified with the 10-marker panel (multiB panel) protocol twice and analyzed on an automatic sequencer (Applied Biosystem ABI 3500 Genetic Analyzer). Results were interpreted using GeneMapper v.6.0. software (Applied Biosystems, USA). Samples that provided no specific PCR products at that stage were discarded. Consensus genotypes were determined using an Access database application programmed by T. Skrbinšek (MisBase, unpublished). Total genotyping success was 47%.

We used Wright's hierarchical structuring of inbreeding (Wright 1931) to estimate the total inbreeding, F_{it}. As suggested by (Keller and Waller 2002), in a randomly breeding population ($F_{is} = 0$) the actual inbreeding that would cause inbreeding depression (probability of alleles at a locus being identical by descent) would equal F_{st} between the studied population and metapopulation / entire species. In the case of Dinaric lynx, because the population has been reintroduced from Slovakian Carpathians, the drift component of inbreeding (F_{st}) directly indicates the inbreeding of the Dinaric population relative to the source population in the Slovakian Carpathians. We used the term '*effective inbreeding (Fe)*' (Frankham et al. 2002), where $Fe = 1 - H_{Din}/H_{SK}$, with H_{Din} being heterozygosity in the Dinaric lynx and H_{SK} being heterozygosity in the source population in Slovakia. We estimated expected heterozygosity of lynx in Slovakia at $He = 0.592$, using the same markers and 60 individuals (including the translocated animals). We used this reference to estimate the dynamics of inbreeding in the Dinaric lynx relative to the original source population in Slovakian Carpathians. As the Alpine stepping-stone subpopulation is currently in its first generation and hence completely outbred, it makes no sense to estimate inbreeding for that subpopulation.

A meta-analysis of inbreeding depression in the wild indicated on average 12 diploid lethal equivalents (2B) in wildlife populations (O'Grady et al. 2006), which matches closely with F-corrected estimates observed in a previous study (Crnokrak and Roff 1999). Using the formula for inbreeding depression $\delta = 1 - e^{-BF}$, where B is the number of gametic lethal equivalents and F the inbreeding coefficient, it is straightforward to estimate the expected inbreeding depression. As we estimated inbreeding relative to the source population in Slovakia, such are also estimates of inbreeding depression. Remaining relative fitness compared to Slovak lynx was calculated as $1 - \delta$.

All translocated lynx were successfully genotyped (N=20), with the exception of two UlyCA2 females, originating from Switzerland, which both dispersed to Austria where one was illegally killed and the fate of the other is unknown (disappeared).

S1.5 Stakeholder engagement, communication and attitude surveys

S1.5.1 Stakeholder engagement and communication with local communities

Stakeholder engagement efforts were primarily directed towards increasing awareness of the precarious conservation status of the lynx population and securing public support for the translocation initiative (Marinko et al. 2020). During the translocation process, LIFE Lynx project placed significant emphasis on building continuous intensive connections with local communities and key stakeholders (i.e. hunters). This included regular communication and one-on-one meetings in the field (mostly in the frame of camera-trapping and GPS tracking of lynx). Special attention was given to highlight the important role played by hunters in the efforts to restore lynx population, including a documentary produced for general public about activities conducted by hunters for support of lynx recovery (https://youtu.be/fayhFYH6P68). In this way we aimed to enhance hunter stewardship for lynx conservation.

Essential part of engaging hunters was through monitoring of the lynx population. All hunting grounds reporting lynx presence opportunistically or through their responses in questionnaires sent each year by every hunting ground in the project area (Fležar et al. 2025) were invited to participate in the survey. In Slovenia, all but one of the contacted hunting grounds responded to the invitation in the first survey year, resulting in camera trapping engaging hunters from the entire lynx distribution in the country. Moreover, the extent of the collaborating hunting grounds grew over the years, following the detected change in lynx distribution (from 36 hunting grounds in 2018-2019 to 63 in 2022-2023). Similarly, the collaboration with hunters and the extent of the survey improved over the years in Croatia. In each collaborating hunting ground, at least one hunter expressed his/her willingness to operate the camera traps during the entire survey period (September to April) on a voluntary basis. In total, more than 100 hunters participated in camera trapping during the 5-year monitoring program in Slovenia, with almost a double number of collaborators within the entire surveyed area including Croatia and Italy (details provided in Fležar et al. 2024, 2025). The LIFE Lynx project personnel trained the hunters to operate the cameras and joined them for camera set up at the start of each survey year. The hunters suggested the optimal sites, checked the cameras on a monthly basis and retrieved the SD cards, and the LIFE Lynx personnel processed the recordings, extracted and identified the lynx and assessed the status of the lynx population on an international level. Besides meeting with the hunters to set up the camera traps and exchange the SD cards, regular feedback from the LIFE Lynx personnel was ensured via publications for hunting magazines, social media, special events and frequent personal communication with every collaborating hunter. Over the years of collaboration, we have established trustful relationships with hunters over most of the lynx distribution in the Dinaric Mountains and SE Alps. With their involvement, hunters have maintained their proactive role in lynx research and conservation, an essential aspect for safeguarding the persistence of the Dinaric SE-Alpine lynx population in the long term.

Hunting organization (Hunters Association of Slovenia) lead the education of law enforcement to prevent the illegal killing of lynx and other wildlife. This included professional training sessions for police officers with the goal of educating them about the importance of detecting, prosecuting, and sanctioning the illegal killings. Moreover, we conducted educational seminars for field personnel (foresters, game wardens and professional hunters), who are most likely to be first to detect and report a suspected illegal killing of wild animals to the police. We also produced a handbook on the investigation of illegal killing of wildlife (Bartol et al. 2019), which was sent to all Slovenian hunters via national hunting magazine *Lovec*.

To facilitate dialogue with local residents in the release areas of Slovenia and Croatia and to strengthen community support for the project, we established Local Consultative Groups (LCGs; Velkavrh et al. 2024). These served as platforms for constructive discussions, addressing local concerns, and co-designing further project activities through regular meetings. Beyond main consultation events, numerous communication activities were implemented within the LCG framework. These aimed to break misconceptions about lynx, provide information on its behavior - particularly in relation to livestock and human interactions compared to the grey wolves and brown bears - and highlight the importance of translocations for lynx conservation. Recognizing that one-way communication often yields limited results, we wanted to create a participatory framework where local communities could actively exchange information, build trust across different interest groups, engage in decision making, and get insight into various aspects of the project. We initialized the process by identifying main stakeholder groups and core messages we wanted to deliver, which were included in the project communication plan (Marinko et al. 2020). Then we reached out to selected individuals and organizations and carried out scoping interviews with them to understand local specifics and ensure that no key stakeholders were unintentionally excluded from the initial LCG meetings. During the first meetings of each LCG group, we introduced project

objectives and activities while also gathering information from the local community to inform planning of future discussion topics. LCGs also provided a space for community members to present their own work related to the lynx conservation. Key themes addressed in the LCG meetings included: implementation and operationalization of the LCGs, collaboration with schools, integration with local tourism initiatives, livestock breeding practices and depredation risks, and engagement with local media. In Slovenia, in additional to the regular local LCG meetings, annual community dinners were introduced from the third year of the project onward. These events brought together members of all LCGs across the country, offering opportunities to share experiences, network, and strengthen community ties. While project team members presented key project outcomes, the program was intentionally kept concise to allow enough time for informal discussions over dinner, as social interaction was recognized as equally important. These events also served as a way to express gratitude to LCG members for their contributions to the project.

S1.5.2 Questionnaire

The main tool for data collection on public attitudes (Dataset2 available via Zenodo at Krofel et al. 2025) was based on a questionnaire including 48 questions (available online in Velkavrh et al. 2024). The process of developing it included identification of the relevant issues to be explored where the entire LIFE Lynx project team has participated in the subsequent design and testing of the wording of the questions. The original questionnaire was designed in English and afterwards participating national teams translated it into local languages (Croatian, Italian, and Slovenian).

S1.5.3 Target groups and sampling

With the public attitude survey, we have targeted the main stakeholder groups which are crucial for lynx conservation – the general public and local hunters in the project area. In Slovenia, a sample of potential general public respondents was obtained from the register of inhabitants – a random stratified (Alps and Dinarics) sample of adult (18 years and older) inhabitants was obtained from the national Statistical Office. The sample included first name, last name, and address of the selected potential respondent. In Italy a commercial panel sample was used and in Croatia a computer-assisted telephone interviewing (CATI) sample. In Croatia, the study was conducted using a CATI telephone method. In Italy questionnaires were filled online or through telephone interviews (Mavec et al. 2024).

Sample of hunters was obtained in Slovenia by sending 3-5 questionnaires to each of the local hunting organizations in the project area and asking the leaders of the hunting organizations to distribute the questionnaires among the hunters. In Croatia a CATI telephone method was carried out and additionally an online survey was shared on various social networks and portals thematically related to the areas of Lika and Gorski Kotar. In Italy a panel survey was carried out and additionally the questionnaire was also distributed by hunters themselves to increase the sample size (Mavec et al. 2024).

Section S2: Results

S2.1 Predation and kleptoparasitism

Overall, the kill rates, prey spectrum and prey consumption (Dataset1, Tables S1 and S2) were similar to the remnant population in the Dinaric mountains studied before (Krofel et al. 2011, 2012, 2013, 2014, 2019; Oliveira et al. 2025), which confirmed their successful integration in the local ecosystems. As the lynx population grows, it will be important to address hunter concerns regarding ungulate abundance and management. The additional predation pressure that the translocated animals, as well as the growing lynx population in general, pose on the prey species, should be appropriately addressed by the ungulate management. To account for this, ungulate management plans in Slovenia were already adjusted with participation of local hunting representatives to allow higher tolerance in reaching harvest quotas for the hunting grounds with confirmed regular lynx presence (Stergar 2024).

Beside direct impact on prey, a large number of scavenger species feeding from prey remains of the translocated lynx indicated widespread trophic interactions, similar to previous studies on the lynx from the remnant population (Krofel and Jerina 2016; Krofel et al. 2012, 2019). Thus, the recovery of this apex predator is increasing the amount of food available to the local scavenger communities and could benefit these species. At the same time, kleptoparasitism by scavengers could reduce the lynx food intake, especially where larger scavengers like brown bears or wild boars are abundant (Duľa and Krofel 2020; Krofel and Jerina 2016; Krofel et al. 2012; Oliveira et al. 2025).

S2.2 Camera trapping

Out of 12 competing models, two were ranked at the top with the difference in AIC < 2 (Table S6). Out of these, the model with highest AIC weight and cumulative weight was chosen as the model with the most support (m9; Table S6).

The baseline detection rate differed between survey years, with highest values being reached in 2021-2022 survey year (Table S7). Moreover, this parameter differed between female vs. male lynx, as well as by the characteristics of a camera trap location type (marking site, road or other location types) (Table S7). It was also evident that the distance at which the females were detected from the center of their presumed home ranges was smaller (3.94 ± 0.22 km) than males (4.98 ± 0.16 km), i.e. indicating that the females have smaller home ranges, which is also confirmed by telemetry data from the region (Dataset1).

The state space encompassed >12,000 km^2 each survey year and remained relatively constant among the survey years (only 6.5% larger state space in 2022-2023 survey year compared to 2019-2020). The standard error of the density and abundance estimates remained almost unchanged over the years, and the bias of the density estimates remained negligible (Efford and Mowat 2014) throughout the survey years. The transience rate ranged between 0.34 (between survey years 2019-2020, 2020-2021 and 2021-2022, 2022-2023), and 0.36 (between survey years 2020-2021, 2021-2022) (Figure S8).

S2.3 Genetics

Altogether, from year 2010 on we collected and analyzed 750 genetic samples from the Dinaric Mountains and Julian Alps (Figure S9), (hair= 301, scat=203, urine=99, saliva from prey=61, blood collected from snow=5, saliva-buccal swab=29, tissue from dead lynx=21, blood samples from captured lynx for telemetry=31). Additionally, we also included already published historical genetic data for the Dinaric lynx population (N=88) obtained through sampling of lynx hunting trophies in Slovenia and Croatia (Polanc et al. 2012; Sindičić et al. 2013).

As both the translocated lynx and their direct offspring are included in the estimates after 2019, we have a strong Wahlund and "isolate breaking" effects (Wahlund 1928) in the final traveling windows. Using expected heterozygosity to estimate inbreeding, we can better understand the effect of the reinforcement on the genetics of the population using empirical data.

According to the last period (40 most recently detected individuals in the dataset), in scenario without the reinforcement, inbreeding would remain high at around Fe=0.32 (Table S8), with inbreeding depression δ = 0.85 (i.e. the fitness of these animals is expected to be 15% of the fitness in the source population). Considering the effects of translocations in the Dinaric mountains with translocated animals and their offspring representing 22.5% of the population (reflecting that ratio in the sample), inbreeding would be

around Fe=0.19 and inbreeding depression $\delta = 0.68$, suggesting more than double increase in fitness compared to the pre-reinforcement. Considering the abundance estimates for the Dinaric part of the population and the numbers of detected offspring (see Results in the main text), we may already be beyond the 22.5% proportion of translocated and F1 lynx in the population. If we include samples from the Alps (scenario of fully connected Alpine stepping-stone and Dinaric Mountains subpopulations) and with the translocated animals and their offspring forming a large part of the population (40% in the sample), inbreeding would drop to 0.08, and fitness increase to four times compared to the pre-reinforcement ($\delta = 0.39$, 61% of the source population fitness). While still high, the estimated inbreeding of Fe=0.08-0.19 is within the range of inbreeding observed in the Dinaric lynx during the 1980s (Sindičić et al. 2013) when the population was still expanding, and fits within the Bonn recommendations (Bonn Lynx Expert Group 2021).

Section S3: Figures

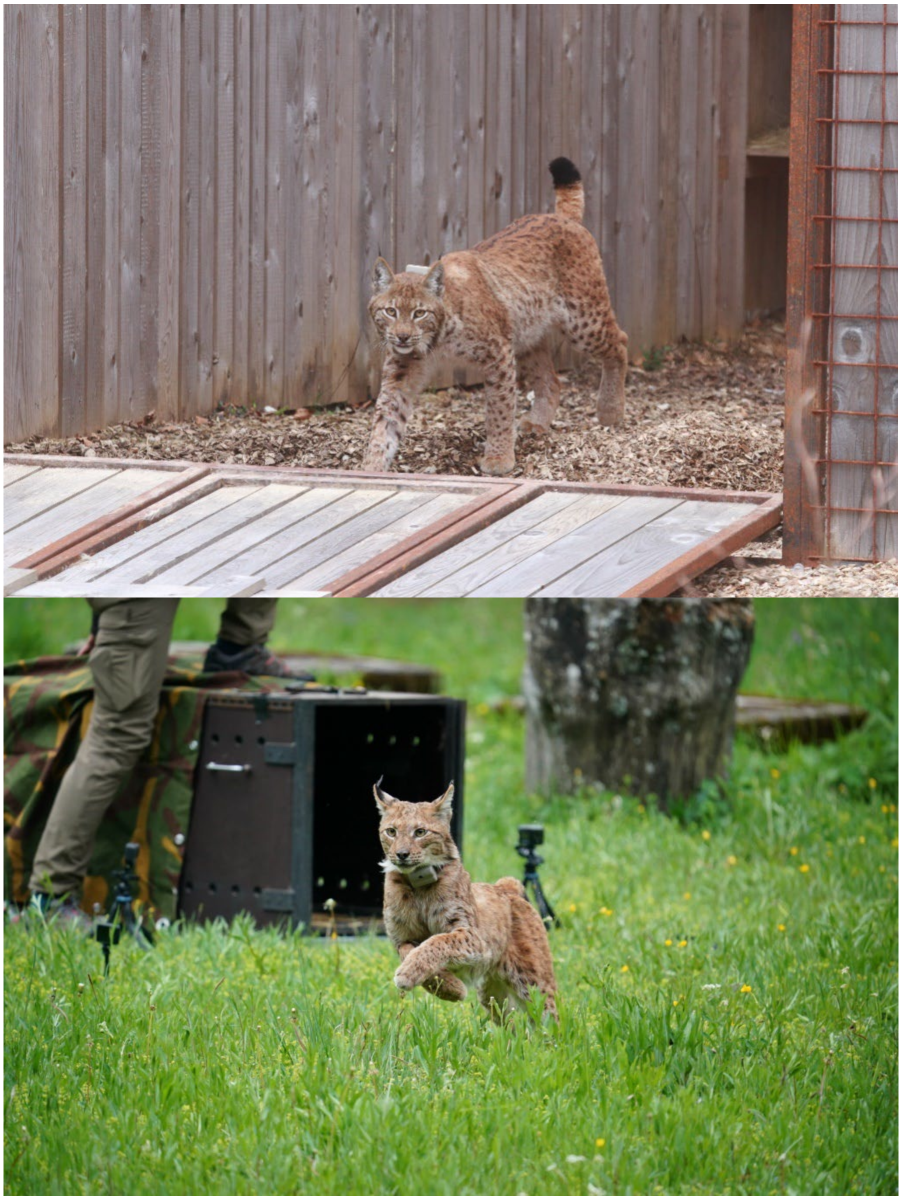

Figure S1. Examples of soft-release (above, Slovenia; photo credit: Miha Krofel) and hard release (below, Croatia; photo credit: Marko Matešić) of lynx.

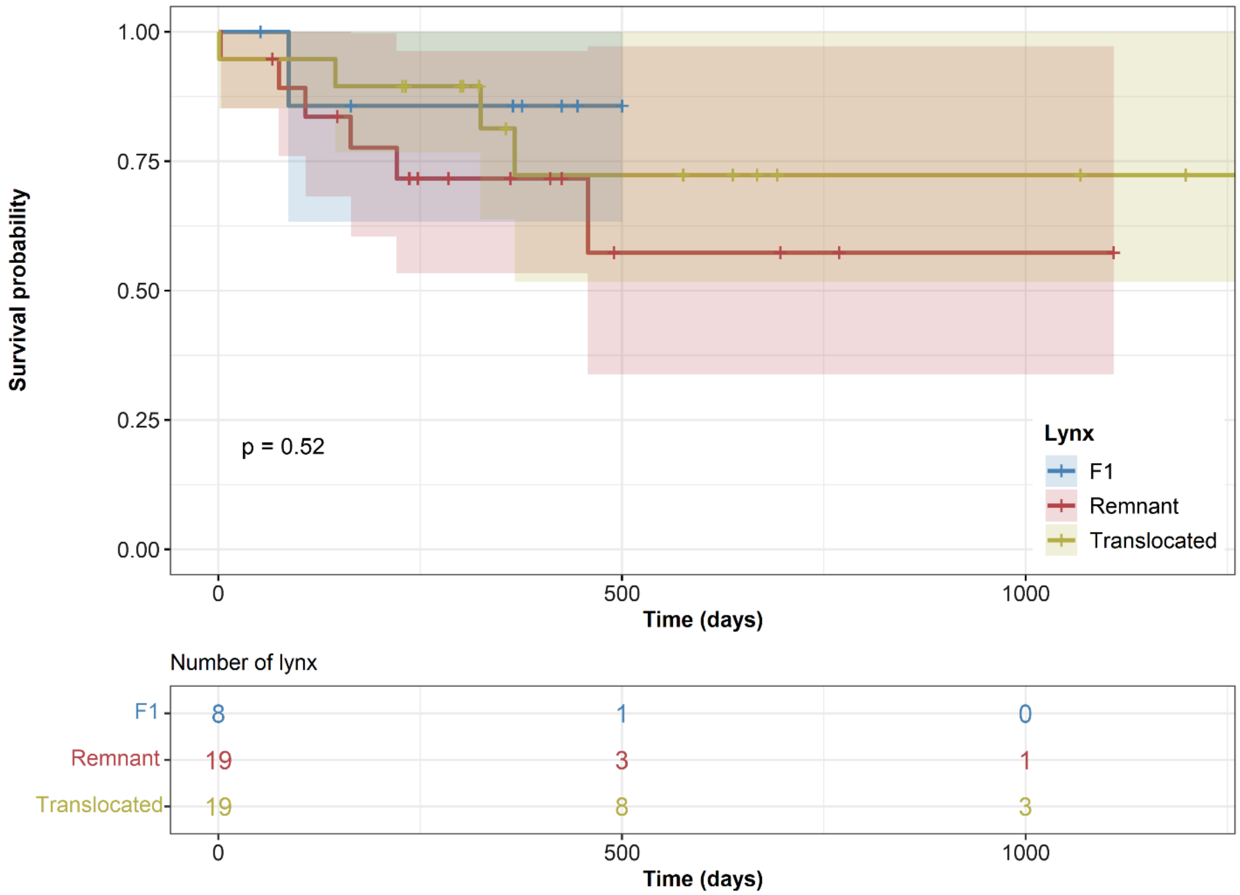

Figure S2. Non-parametric survival estimates for lynx within the tracking period. Product-limit (Kaplan-Meier) survival estimates for remnant, translocated, and F1 lynx. Lynx with unknown status at the end of the tracking period were excluded from the analysis.

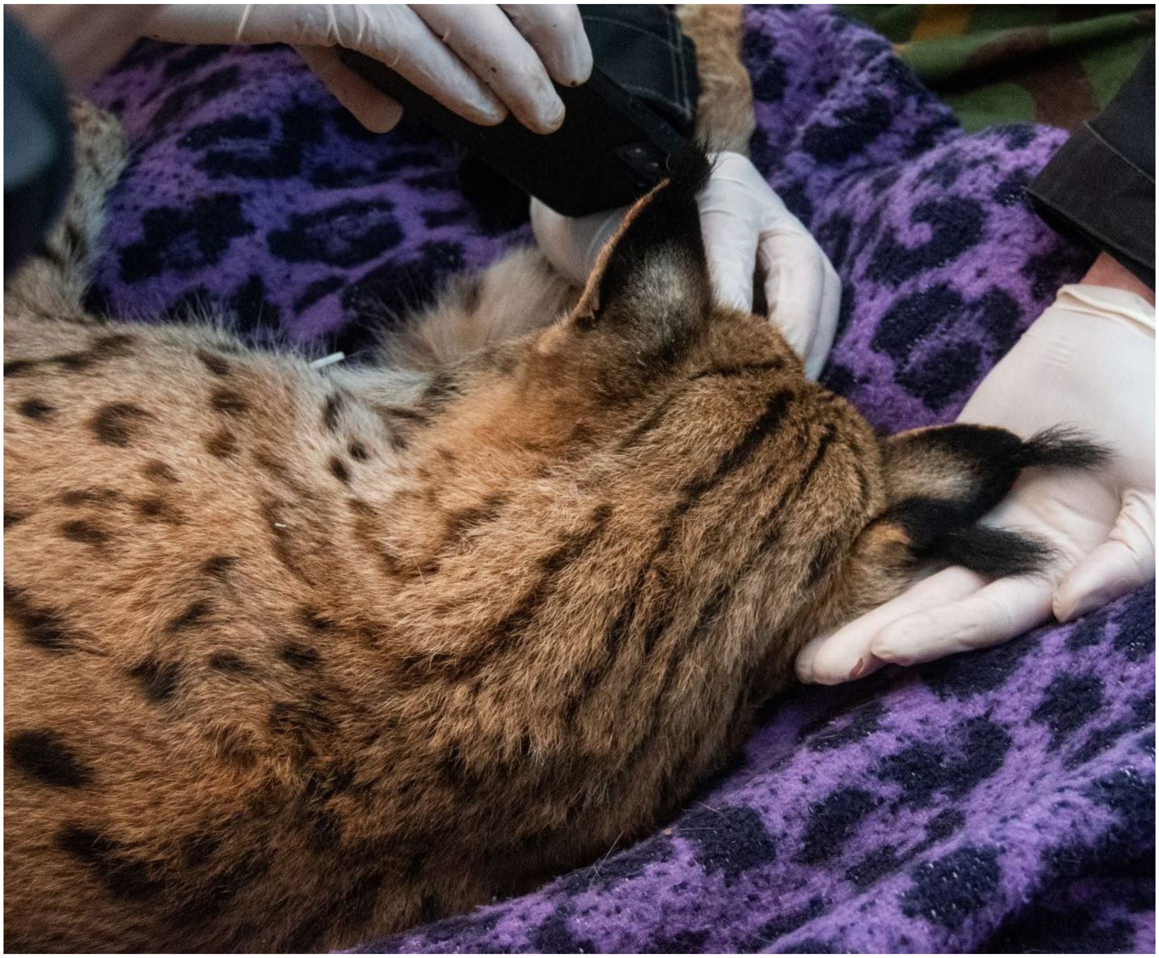

Figure S3. Example of a morphological deformation in a male lynx from the remnant population in Slovenia (double ear-tufts on the right ear), possibly associated with inbreeding (photo credit: Maj Hočevar).

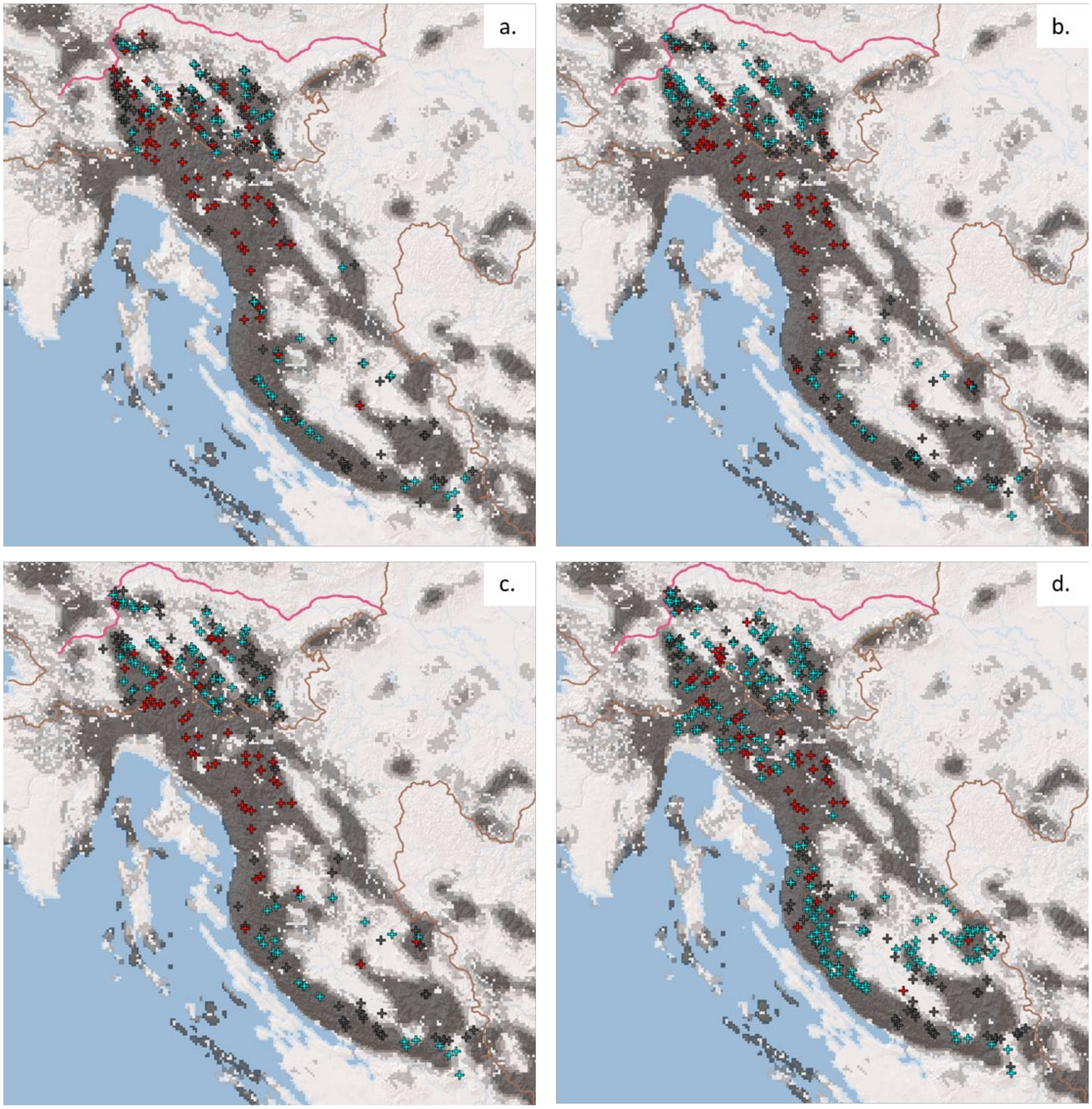

Figure S4. The locations of camera traps in Northern Dinaric Mountains in each survey year (a: 2019-2020, b: 2020-2021, c: 2021-2022, d: 2022-2023). The colors of crosses show different types of location; red – marking sites, blue – roads and grey – other types of locations. Lynx habitat suitability model (Skrbinšek and Krofel 2008) is included in the maps (grey shading).

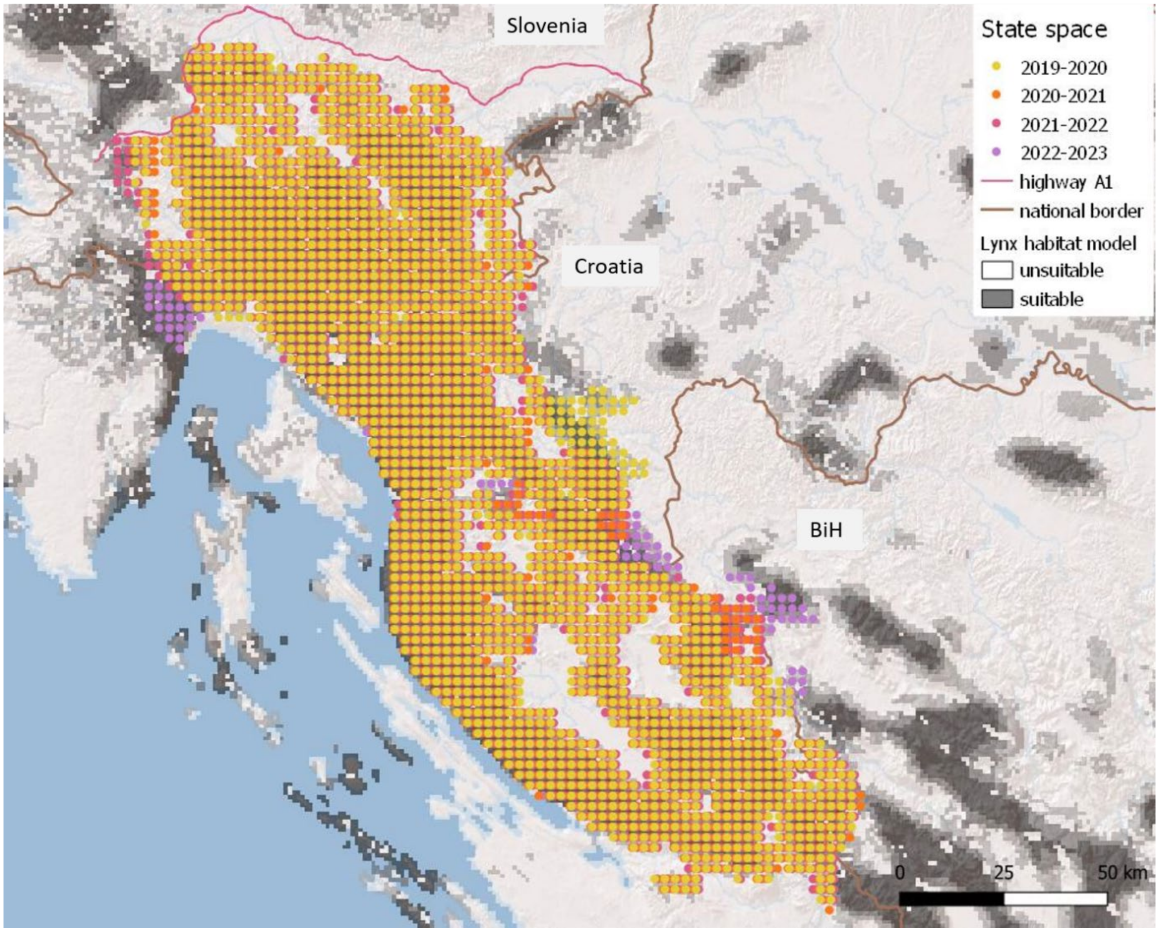

Figure S5. The state space for the four survey years shown together with the habitat suitability model (Skrbinšek and Krofel 2008) and the A1 highway in Slovenia.

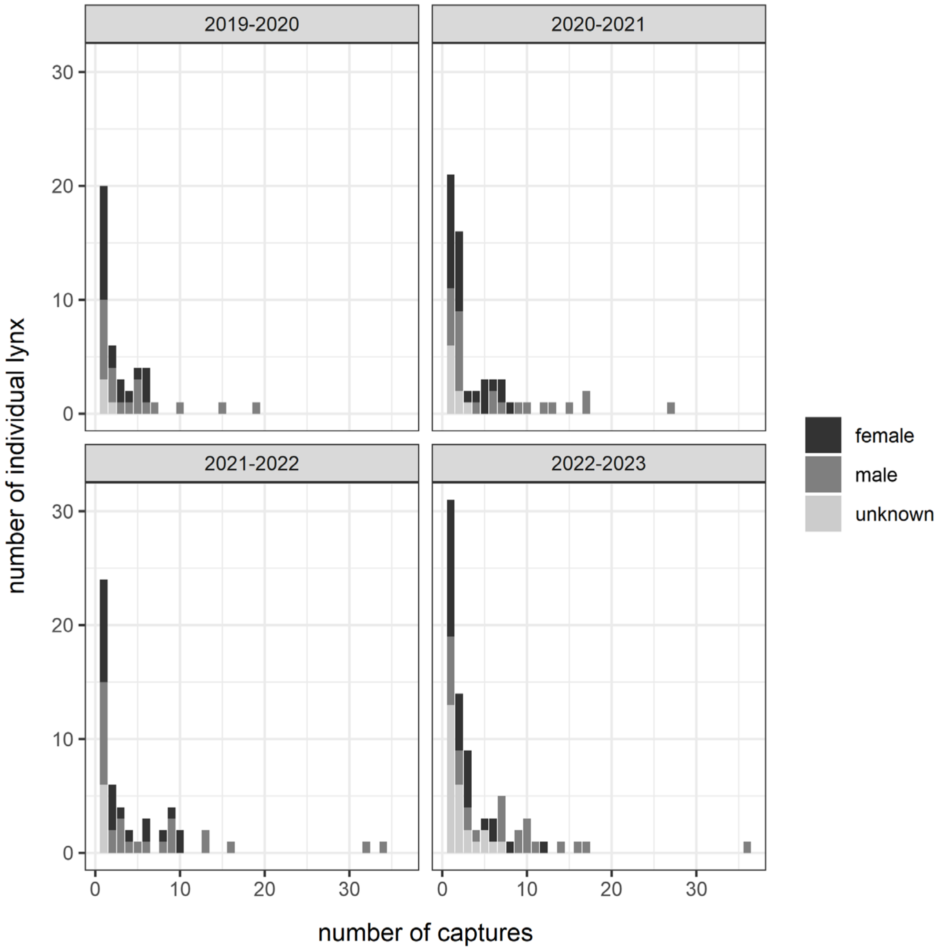

Figure S6. Lynx recaptures during each survey year; the maximum number of recaptures (n=50) was recorded for a male in 2022-2023.

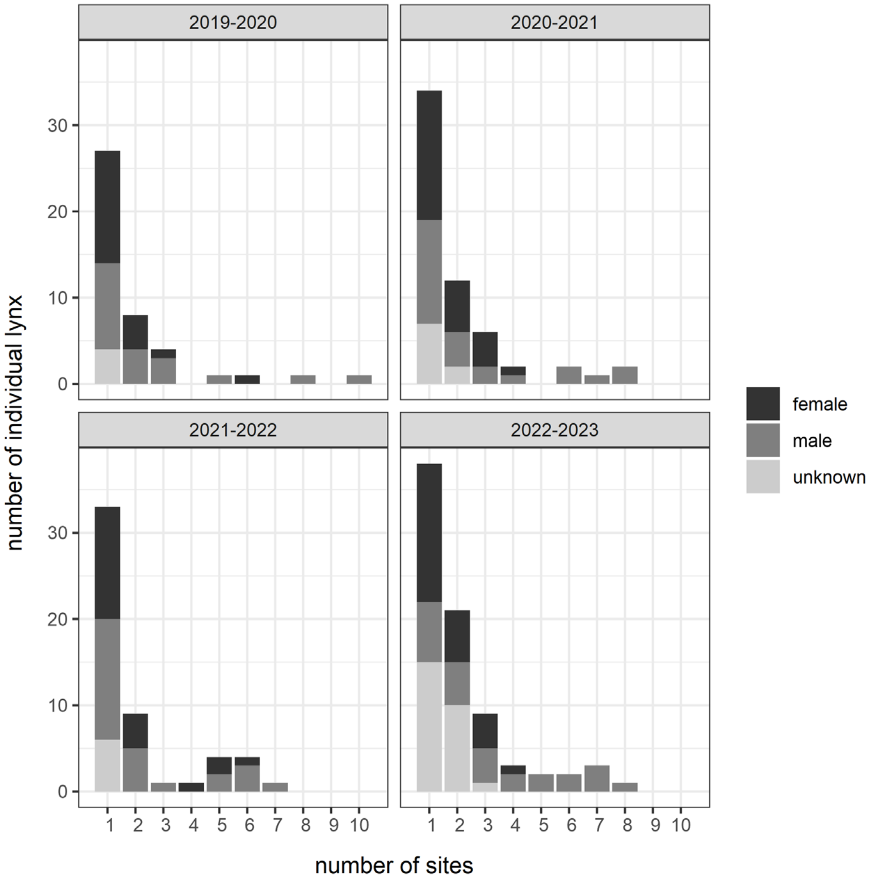

Figure S7. Lynx spatial recaptures during each survey year; the maximum number of spatial recaptures (n=11) was recorded for a male in 2022-2023.

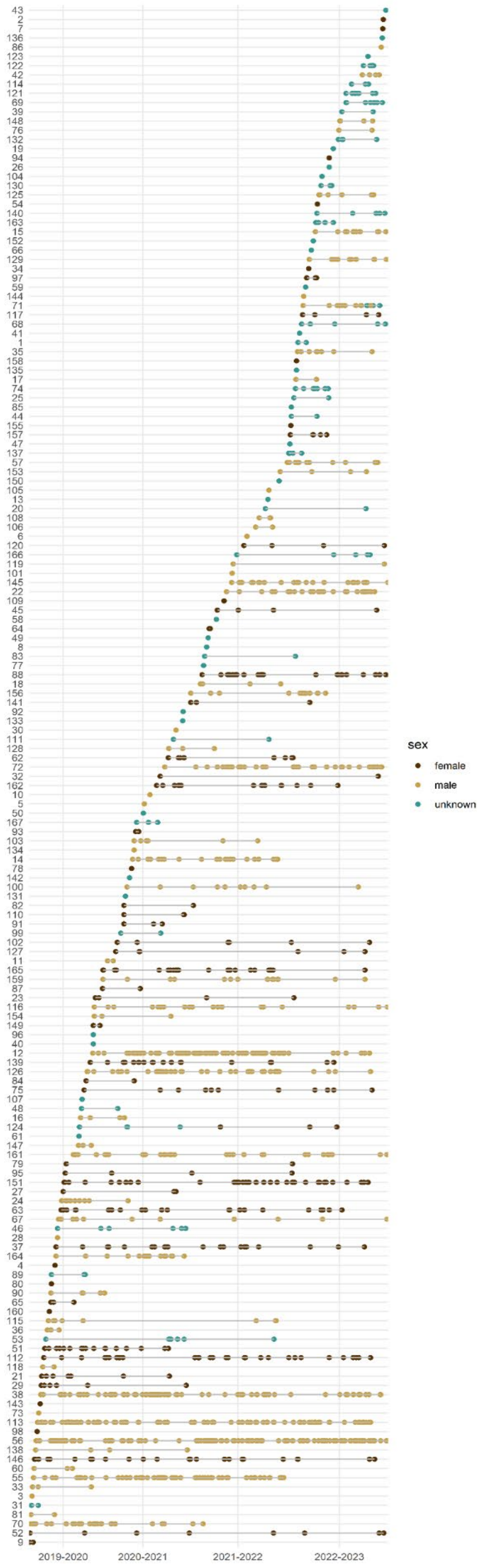

Figure S8. Transience of individual lynx between the survey years. The start of each survey year is marked on the x axis while the individual lynx are shown as numbers on the y axis.

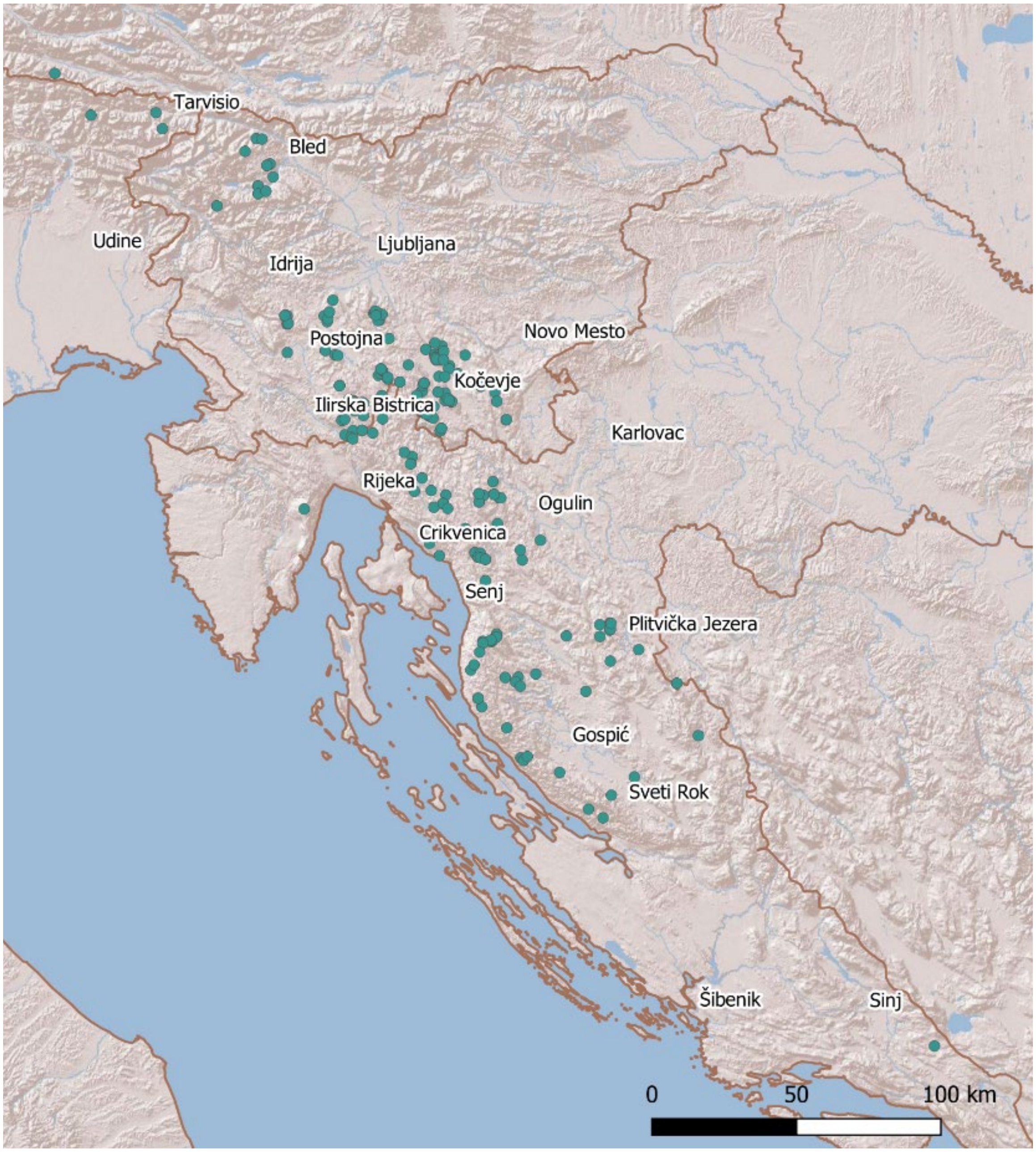

Figure S9. Locations of successfully genotyped genetic samples (collected between 2010-2024), where individual lynx were identified.

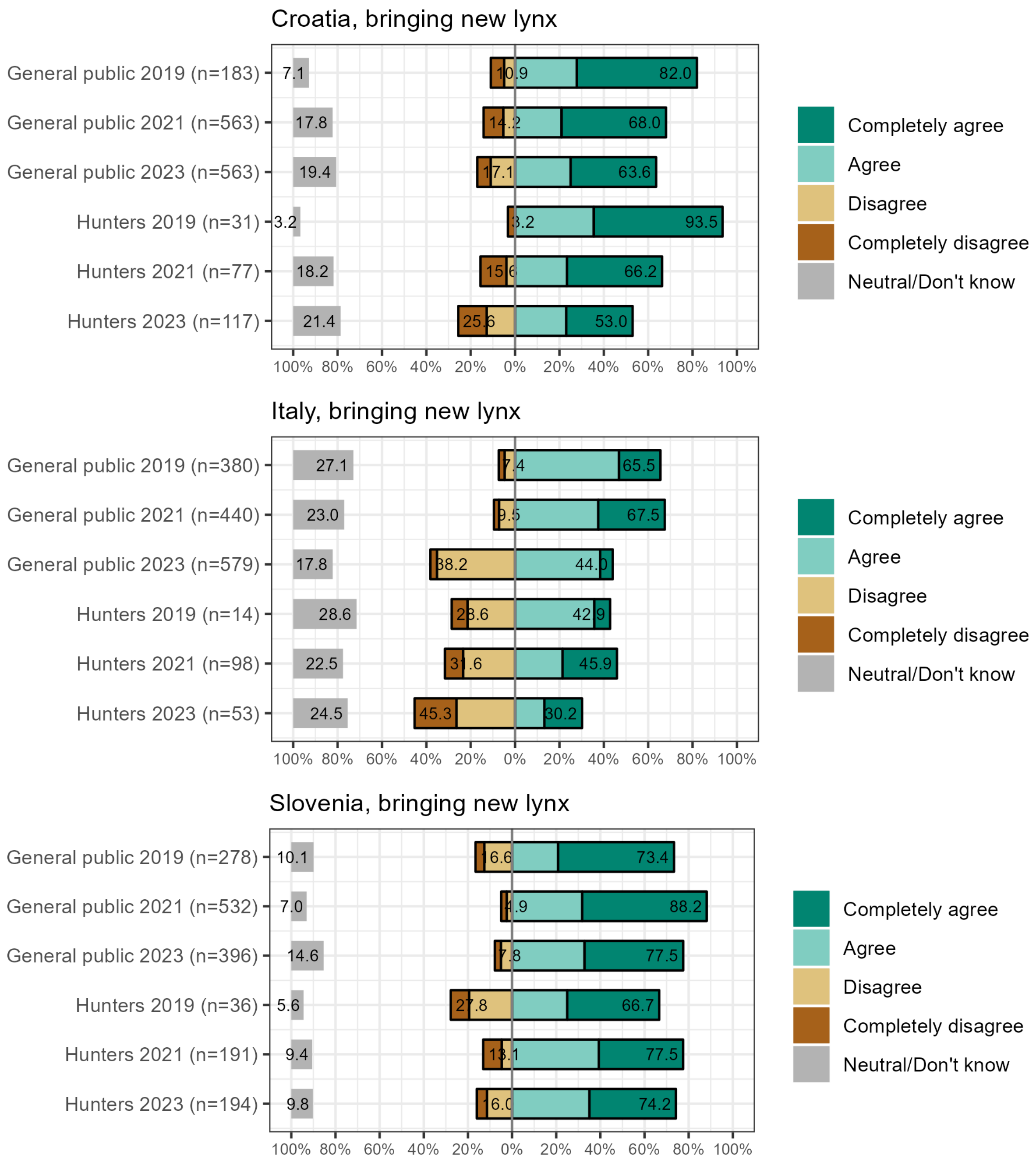

Figure S10. Responses to the statement: "I support bringing new lynx to Croatia / Italy / Slovenia to save the population". Respondents answered for their respective countries.

Section S4: Tables

Table S1. List of vertebrate scavenger species recorded feeding on kills of translocated lynx monitored with video surveillance in the Dinaric Mountains and Alps in Slovenia (n=62). Lynx were recorded as scavenging, when another lynx was recorded feeding on a kill made by translocated lynx (prey sharing).

Common name	Latin name
Brown bear	*Ursus arctos*
Grey wolf	*Canis lupus*
Golden jackal	*Canis aureus*
Red fox	*Vulpes vulpes*
Feral dog	*Canis lupus familiaris*
Eurasian lynx (scavenging)	*Lynx lynx*
European Wildcat	*Felis silvestris*
Eurasian badger	*Meles meles*
Beech marten	*Martes foina*
Pine marten	*Martes martes*
Wildboar	*Sus scrofa*
Bank vole	*Myodes glareolus*
Golden eagle	*Aquila chrysaetos*
White-tailed eagle	*Haliaeetus albicilla*
Common buzzard	*Buteo buteo*
Eurasian goshawk	*Accipiter gentilis*
Common raven	*Corvus corax*
Eurasian jay	*Garrulus glandarius*
Coal tit	*Parus ater*

Table S2. List and proportion of prey species recorded at kill sites of translocated lynx in Italy, Croatia and Slovenia (n=253).

**Common name**	**Latin name**	**Proportion among kills (%)**
Roe deer	*Capreolus capreolus*	83.4
Red deer	*Cervus elaphus*	8.7
Chamois	*Rupicapra rupicapra*	3.6
Red fox	*Vulpes vulpes*	1.2
Mouflon	*Ovis ammon*	0.8
Brown hare	*Lepus europaeus*	0.4
European badger	*Meles meles*	0.4
European wildcat	*Felis silvestris*	0.4
Unknown	-	1.2

Table S3. Mean female, male and total densities estimates with standard errors and 95% confidence intervals (in brackets) over four survey years for session-specific state spaces. The precision of the estimate is shown with coefficient of variation (Efford and Mowat 2014).

	**2019-2020**	**2020-2021**	**2021-2022**	**2022-2023**
**population density**	0.88 ± 0.15 (0.63-1.23)	1.05 ± 0.15 (0.80-1.38)	0.91 ± 0.13 (0.68-1.20)	1.27 ± 0.15 (1.00-1.61)
**female density**	0.61 ± 0.12 (0.42-0.88)	0.72 ± 0.12 (0.52-1.00)	0.62 ± 0.10 (0.45-0.86)	0.87 ± 0.13 (0.65-1.16)
**male density**	0.28 ± 0.05 (0.20-0.40)	0.33 ± 0.05 (0.25-0.45)	0.28 ± 0.05 (0.21-0.39)	0.40 ± 0.06 (0.31-0.53)
**population abundance**	110 ± 18 (79-152)	129 ± 18 (98-169)	112 ± 16 (84-149)	156 ± 19 (123-198)
**coefficient of variation**	0.179	0.156	0.162	0.141
**state space (km^2)**	12,350	12,206	12,350	12,275

Table S4. Camera trapping effort per survey year shown as i) number of camera trapping sites set on marking sites, roads and other type of locations in the Dinaric Mts., ii) cumulative number of and observed mean distance (km) between camera trapping sites (Nearest neighbor analysis tool, QGIS v. 3.6.0) per country (Slovenia, Croatia) and in total study area (Dinaric Mts) and iii) the cumulative number of camera trapping days for the Dinaric Mts.

		2019-2020	2020-2021	2021-2022	2022-2023
**Number of camera trapping sites**	**Marking site**	53	56	51	47
	**Road**	64	76	79	171
	**Other**	123	107	95	111
	**Slovenia**	146	131	129	119
	**Croatia**	94	108	96	210
	**Dinaric Mts.**	240	239	225	329
**Mean distance between camera trapping sites**	**Slovenia**	1.08	1.29	1.40	1.47
	**Croatia**	2.34	2.25	2.52	1.80
	**Dinaric Mts.**	2.28	2.38	2.61	2.34
**Number of camera trapping days**	**Dinaric Mts.**	24,874	28,142	29,069	34,817

Table S5. Overview of lynx camera-trap captures for the entire study area (Dinaric Mountains) per survey year. Each survey year corresponds to a survey period which lasted from Aug 15th to Feb 15th.

		2019-2020	2020-2021	2021-2022	2022-2023
**Total spatial recaptures**		84	119	109	170
**Total recaptures**		148	257	275	341
**Mean recaptures**		3.44	4.36	5.19	4.32
**Mean spatial recaptures**		1.95	2.02	2.06	2.15
**MMDM**		10.71	9.14	10.23	8.63
**Number of individual lynx**	*females*	19	26	21	27
	males	20	24	26	26
	unknown sex	4	9	6	26
	total	43	59	53	79

Table S6. The selection of the 12 competing models. Model structure shows the effects tested per parameter, and AIC, AIC weight and cumulative weight are given for each competing model. The top-ranking models are bolded while the most supported model is additionally in italics. Session represents survey year.

model structure	model ID	AIC	AIC weight	Cumulative weight
D~session, p0~1, sig~1	m0	12978	4.90E-168	1
D~session, p0~session, sig~session	m1	12966	1.80E-165	1
D~session, p0~session+b, sig~session	m2	12435	3.00E-50	1
D~session, p0~session+sex, sig~session	m3	12745	1.90E-117	1
D~session, p0~session+b+sex, sig~session	m4	12298	1.50E-20	1
D~session, p0~session+location_type, sig~session	m5	12831	2.80E-136	1
D~session, p0~session+b+location_type, sig~session	m6	12360	8.50E-34	1
D~session, p0~session+sex+location_type, sig~session	m7	12628	4.60E-92	1
D~session, p0~session+b+sex+location_type, sig~session	m8	12226	7.50E-05	1
D~session, p0~session+b+sex+location_type, sig~sex	***m9***	***12210***	***2.80E-01***	***0.96***
D~session, p0~session+b+sex+location_type, sig~session+sex	m10	12214	3.50E-02	1
D~session, p0~session+b+sex+location_type, sig~1	m11	12221	9.40E-04	1
**D~session, p0~b+sex+location_type, sig~sex**	**m12**	**12208**	**6.80E-01**	**0.68**

Table S7. Baseline detection rate across different survey years and types of camera trap location, given per lynx sex with standard error (SE) and 95% confidence interval.

Survey year	Sex	Location type	Estimate	SE	95% CI
2019-2020	f	Marking site	0.004	0.001	0.003-0.006
2019-2020	f	Road	0.002	0.000	0.002-0.003
2019-2020	f	Other	0.002	0.000	0.001-0.002
2019-2020	m	Marking site	0.010	0.002	0.007-0.014
2019-2020	m	Road	0.006	0.001	0.004-0.008
2019-2020	m	Other	0.004	0.001	0.003-0.006
2020-2021	f	Marking site	0.005	0.001	0.003-0.006
2020-2021	f	Road	0.003	0.000	0.002-0.004
2020-2021	f	Other	0.002	0.000	0.001-0.003
2020-2021	m	Marking site	0.011	0.001	0.009-0.015
2020-2021	m	Road	0.007	0.001	0.005-0.009
2020-2021	m	Other	0.005	0.001	0.004-0.006
2021-2022	f	Marking site	0.005	0.001	0.004-0.007
2021-2022	f	Road	0.003	0.000	0.002-0.004
2021-2022	f	Other	0.002	0.000	0.002-0.003
2021-2022	m	Marking site	0.013	0.002	0.010-0.016
2021-2022	m	Road	0.008	0.001	0.006-0.010
2021-2022	m	Other	0.005	0.001	0.004-0.007
2022-2023	f	Marking site	0.004	0.001	0.003-0.006
2022-2023	f	Road	0.003	0.000	0.002-0.003
2022-2023	f	Other	0.002	0.000	0.001-0.002
2022-2023	m	Marking site	0.010	0.001	0.008-0.014
2022-2023	m	Road	0.006	0.001	0.005-0.008
2022-2023	m	Other	0.004	0.001	0.003-0.006

Table S8. Genetic diversity parameters estimated at the end of the translocation process (year 2024). Remnant Dinaric: calculated without the translocated lynx and their offspring, exploring situation without the effect of translocations; Dinaric w/Reinforcement: including lynx translocated to Dinaric Mountains and their offspring; Including Alp Step. St.: with all translocated lynx, including the Alpine stepping stone subpopulation. The "Reinf." Value indicates the proportion of translocated animals and their offspring in the final traveling window.

	**A**	**SEA**	**Ho**	**SEHo**	**He**	**SEHe**	**Fe**	**SEFe**
Remnant Dinaric	2.79	0.17	0.40	0.04	0.40	0.03	0.32	0.06
Dinaric w/Reinforcement (Reinf. = 22.5%)	3.58	0.30	0.43	0.04	0.48	0.03	0.19	0.05
Including Alp Step. St. (Reinf. = 40%)	3.89	0.35	0.47	0.03	0.54	0.03	0.08	0.04

Section S5: References

Adams, J.R., Waits, L.P., 2007. An efficient method for screening faecal DNA genotypes and detecting new individuals and hybrids in the red wolf (*Canis rufus*) experimental population area. Conservation Genetics 8, 123-131.

Allen, M.L., Hočevar, L., de Groot, M., Krofel, M., 2017. Where to leave a message? The selection and adaptive significance of scent-marking sites for Eurasian lynx. Behavioral Ecology and Sociobiology 71, 136.

Andrén, H., Linnell, J.D.C., Liberg, O., Andersen, R., Danell, A., Karlsson, J., Odden, J., Moa, P.F., Ahlqvist, P., Kvam, T., Franzén, R., Segerström, P., 2006. Survival rates and causes of mortality in Eurasian lynx (*Lynx lynx*) in multi-use landscapes. Biological Conservation 131, 23-32.

Bartol, M., Belaj, U., Blažič, M., Černe, R., Fležar, U., Hudoklin, A., Oven, N., Pagon, N., Potočnik, H., Praček, R., Prešeren, N., Rot, A., Skrbinšek, T., Smolej, T., Stergar, M., Vengušt, G., Vrček, D., Žele Vengušt, D., Žerjav, S., 2019. The Handbook for Investigation of Poaching. Hunting Association of Slovenia, Ljubljana.

Bonn Lynx Expert Group, 2021. Recommendations for the conservation of the Eurasian lynx in Western and Central Europe. Cat News Special Issue 14, 78-87.

Burnham, K.P., Anderson, D.R., 2004. Multimodel Inference: Understanding AIC and BIC in Model Selection. Sociological Methods & Research 33, 261-304.

Choo, Y.R., Kudavidanage, E.P., Amarasinghe, T.R., Nimalrathna, T., Chua, M.A.H., Webb, E.L., 2020. Best practices for reporting individual identification using camera trap photographs. Global Ecology and Conservation 24, e01294.

Crnokrak, P., Roff, D.A., 1999. Inbreeding depression in the wild. Heredity 83, 260-270.

Duľa, M., Bojda, M., Chabanne, D.B.H., Drengubiak, P., Hrdý, Ľ., Krojerová-Prokešová, J., Kubala, J., Labuda, J., Marčáková, L., Oliveira, T., Smolko, P., Váňa, M., Kutal, M., 2021. Multi-seasonal systematic camera-trapping reveals fluctuating densities and high turnover rates of Carpathian lynx on the western edge of its native range. Scientific Reports 11, 9236.

Duľa, M., Krofel, M., 2020. A cat in paradise: hunting and feeding behaviour of Eurasian lynx among abundant naive prey. Mammalian Biology.

Dupont, G., Royle, J.A., Nawaz, M.A., Sutherland, C., 2021. Optimal sampling design for spatial capture–recapture. Ecology 102, e03262.

Efford, M.G., Dawson, D.K., Robbins, C.S., 2004. DENSITY: software for analysing capture-recapture data from passive detector arrays. Animal Biodiversity and Conservation 27, 217-228.

Efford, M.G., Fewster, R.M., 2013. Estimating population size by spatially explicit capture–recapture. Oikos 122, 918-928.

Efford, M.G., Mowat, G., 2014. Compensatory heterogeneity in spatially explicit capture–recapture data. Ecology 95, 1341-1348.

Fležar, U., Aronsson, M., Černe, R., Pičulin, A., Bartol, M., Stergar, M., Rot, A., Hočevar, L., Topličanec, I., Sindičić, M., Gomerčić, T., Slijepčević, V., Krofel, M., 2023. Using heterogeneous camera-trapping sites to obtain the first density estimates for the transboundary Eurasian lynx (*Lynx lynx*) population in the Dinaric Mountains. Biodiversity and Conservation 32, 3199-3216.

Fležar, U., Hočevar, L., Sindičić, M., Gomerčić, T., Konec, M., Slijepčević, V., Bartol, M., Boljte, B., Črtalič, J., Jelenčič, M., Kljun, F., Molinari-Jobin, A., Pičulin, A., Potočnik, H., Rot, A., Skrbinšek, T., Topličanec, I., Černe, R., Krofel, M., 2024. Surveillance of the reinforcement process of the Dinaric - SE Alpine lynx population in the lynx-monitoring year 2022-2023. LIFE Lynx project report, Ljubljana. https://www.lifelynx.eu/wp-content/uploads/2024/04/LIFE-Lynx-C5-annual-report_FINAL.pdf

Fležar, U., Krofel, M., Bartol, M., Černe, R., Trajbarič, A., Hvala, T., Stergar, M., Aronsson, M., 2025. Hunter-engaged monitoring of the Eurasian lynx during the reinforcement process. Wildlife Biology e01399.

Frankham, R., Ballou, J.D., Briscoe, D.A., 2002. Introduction to Conservation Genetics. Cambridge University Press, Cambridge.

Hendry, H., Mann, C., 2017. Camelot – Intuitive Software for Camera Trap Data Management. bioRxiv, 203216.

Johnson, J.B., Omland, K.S., 2004. Model selection in ecology and evolution. Trends in Ecology & Evolution 19, 101-108.

Keller, L.F., Waller, D.M., 2002. Inbreeding effects in wild populations. Trends in Ecology & Evolution 17, 230-241.
Krofel, M., Fležar, U., Černe, R., Hočevar, L., Konec, M., Majić Skrbinšek, A., Skrbinšek, T., Wilson, S., Bele, B., Črtalič, J., Gomerčić, T., Hvala, T., Kubala, J., Kvapil, P., Mavec, M., Molinari-Jobin, A., Molinari, P., Pazhenkova, E., Potočnik, H., Sin, T., Sindičić, M., Topličanec, I., Oliveira, T., 2025. Dinaric-Alpine lynx recovery and attitude surveys datasets (v1.2). Zenodo. https://doi.org/10.5281/zenodo.15252332
Krofel, M., Fležar, U., Hočevar, L., Sindičić, M., Gomerčić, T., Konec, M., Slijepčević, V., Bartol, M., Boljte, B., Črtalič, J., Jelenčič, M., Kljun, F., Molinari-Jobin, A., Pičulin, A., Potočnik, H., Rot, A., Skrbinšek, T., Topličanec, I., Černe, R., 2021. Surveillance of the reinforcement process of the Dinaric - SE Alpine lynx population in the lynx-monitoring year 2019-2020. Technical report., p. 45. University of Ljubljana, Biotechnical Faculty, Ljubljana.
Krofel, M., Huber, D., Kos, I., 2011. Diet of Eurasian lynx *Lynx lynx* in the northern Dinaric Mountains (Slovenia and Croatia): Importance of edible dormouse *Glis glis* as alternative prey. Acta Theriologica 56, 315-322.
Krofel, M., Jerina, K., 2016. Mind the cat: Conservation management of a protected dominant scavenger indirectly affects an endangered apex predator. Biological Conservation 197, 40-46.
Krofel, M., Jerina, K., Kljun, F., Kos, I., Potočnik, H., Ražen, N., Zor, P., Žagar, A., 2014. Comparing patterns of human harvest and predation by Eurasian lynx *Lynx lynx* on European roe deer *Capreolus capreolus* in a temperate forest. European Journal of Wildlife Research 60, 11-21.
Krofel, M., Kos, I., Jerina, K., 2012. The noble cats and the big bad scavengers: effects of dominant scavengers on solitary predators. Behavioral Ecology and Sociobiology 66, 1297-1304.
Krofel, M., Potočnik, H., Skrbinšek, T., Kos, I., 2006. Movement and predation patterns of Eurasian lynx (*Lynx lynx*) on Menišija and Logatec plateau (Slovenia). Veterinarske novice 32, 11-17.
Krofel, M., Skrbinšek, T., Kos, I., 2013. Use of GPS location clusters analysis to study predation, feeding, and maternal behavior of the Eurasian lynx. Ecological Research 28, 103-116.
Krofel, M., Skrbinšek, T., Mohorović, M., 2019. Using video surveillance to monitor feeding behaviour and kleptoparasitism at Eurasian lynx kill sites. Journal of Vertebrate Biology 68, 55-65, 11.
Kubala, J., Smolko, P., Klinga, P., Tám, B., Kropil, R., 2018. Monitoring protocol for the Eurasian lynx (*Lynx lynx*) population in the Slovak Carpathian Mountains. LIFE Lynx project report. , Zvolen.
Marinko, U., Velkavrh, M., Molinari Jobin, A., Nanni, V., Sindičić, M., Sever, M., Majić Skrbinšek, A., 2020. LIFE Lynx Communication Manual. LIFE Lynx project report, Ljubljana.
Mavec, M., Skrbinšek, T., Majić Skrbinšek, A., Bele, B., Molinari Jobin, A., Selanec, I., 2024. Assessment of public attitudes toward lynx and lynx conservation in Slovenia, Croatia and Italy. LIFE Lynx project report, Ljubljana. https://www.lifelynx.eu/wp-content/uploads/2024/04/D4_final-report_popravki.pdf
Menotti-Raymond, M., David, V.A., Lyons, L.A., Schäffer, A.A., Tomlin, J.F., Hutton, M.K., O'Brien, S.J., 1999. A genetic linkage map of microsatellites in the domestic cat (*Felis catus*). Genomics 57, 9-23.
Menotti-Raymond, M.A., David, V.A., Wachter, L.L., Butler, J.M., O'Brien, S.J., 2005. An STR forensic typing system for genetic individualization of domestic cat (*Felis catus*) samples. Journal of Forensic Sciences 50, 1061-1070.
O'Grady, J.J., Brook, B.W., Reed, D.H., Ballou, J.D., Tonkyn, D.W., Frankham, R., 2006. Realistic levels of inbreeding depression strongly affect extinction risk in wild populations. Biological Conservation 133, 42-51.
Oliveira, T., Carricondo-Sanchez, D., Mattisson, J., Vogt, K., Corradini, A., Linnell, J.D.C., Odden, J., Heurich, M., Rodríguez-Recio, M., Krofel, M., 2023. Predicting kill sites of an apex predator from GPS data in different multiprey systems. Ecological Applications 33, e2778.
Oliveira, T., Mattisson, J., Vogt, K., Linnell, J., Odden, J., Oeser, J., Premier, J., Rodríguez-Recio, M., Belotti, E., Bufka, L., Černe, R., Duľa, M., Fležar, U., Gonev, A., Herdtfelder, M., Heurich, M., Hočevar, L., Hvala, T., Iľko, T., Kont, R., Koubek, P., Krojerová-Prokešová, J., Kubala, J., Kübarsepp, M., Kusak, J., Kutal, M., Machciník, B., Männil, P., Melovski, D., Molinari, P., Ornicāns, A., Pavlov, A., Prostor, M., Slijepčević, V., Smolko, P., Tam, B., Krofel, M., 2025. Ecological and intrinsic drivers of foraging parameters of Eurasian lynx at a continental scale. Journal of Animal Ecology 94, 154-167.
Polanc, P., Sindičić, M., Jelenčič, M., Gomerčić, T., Kos, I., Huber, Đ., 2012. Genotyping success of historical Eurasian lynx (*Lynx lynx* L.) samples. Molecular Ecology Resources 12, 293-298.
Royle, J.A., Chandler, R.B., Sollmann, R., Gardner, B., 2013. Spatial Capture-Recapture. Elsevier Science.

Sin, T., Marocco, I., Kraft, B., Pop, I.M., Gazzola, A., 2021. Assessment and selection of sites and lynx for live-capture from the Carpathian source population in Romania. LIFE Lynx project report. , Focşani.
Sindičić, M., Polanc, P., Gomerčić, T., Jelenčič, M., Huber, Đ., Trontelj, P., Skrbinšek, T., 2013. Genetic data confirm critical status of the reintroduced Dinaric population of Eurasian lynx. Conservation Genetics 14, 1009-1018.
Skrbinšek, T., 2017. Collecting lynx noninvasive genetic samples. Instruction manual for field personnel and volunteers. Ljubljana.
Skrbinšek, T., Krofel, M., 2008. Analiza kvalitete habitata, hrana in kompeticija. Project DinaRis report. Oddelek za biologijo, Biotehniška fakulteta, Univerza v Ljubljani, Ljubljana.
Stergar, M., 2024. Kaj nam prinašajo lovskoupravljavski načrti za obdobje 2021–2030? Lovec 107, 112-115.
Stergar, M., Slijepčević, V., 2017. Lynx camera trapping guidelines. LIFE Lynx project report., Ljubljana.
Sutherland, C., Royle, J.A., Linden, D.W., 2019. oSCR: a spatial capture–recapture R package for inference about spatial ecological processes. Ecography 42, 1459-1469.
Taberlet, P., Griffin, S., Goossens, B., Questiau, S., Manceau, V., Escaravage, N., Waits, L.P., Bouvet, J., 1996. Reliable genotyping of samples with very low DNA quantities using PCR. Nucleic Acids Research 24, 3189-3194.
Velkavrh, M., Kavčič, I., Topličanec, I., Majić Skrbinšek, A., 2024. Our experience in working with stakeholders through Local consultative groups. LIFE Lynx project report, Ljubljana. https://www.lifelynx.eu/wp-content/uploads/2024/04/Our-experience-in-working-with-stakeholders-through-Local-consultative-groups_Life-Lynx.pdf
Wahlund, S., 1928. Zuzammensetzung von populationen und korrelation-serscheiunung von standpunkt der vererbungslehre aus betrachtet. Hereditas 11, 65–106.
Williamson, J.E., Huebinger, R.M., Sommer, J.A., Louis Jr, E.E., Barber, R.C., 2002. Development and cross-species amplification of 18 microsatellite markers in the Sumatran tiger (*Panthera tigris sumatrae*). Molecular Ecology Notes 2, 110-112.
Wright, S., 1931. Evolution in Mendelian Populations. Genetics 16, 97-159.
